# Post-Transcriptional Regulation of the Trypanosome Heat Shock Response by a Zinc Finger Protein

**DOI:** 10.1371/journal.ppat.1003286

**Published:** 2013-04-04

**Authors:** Dorothea Droll, Igor Minia, Abeer Fadda, Aditi Singh, Mhairi Stewart, Rafael Queiroz, Christine Clayton

**Affiliations:** Zentrum für Molekulare Biologie der Universität Heidelberg (ZMBH), DKFZ-ZMBH Alliance, Heidelberg, Germany; Yale University, United States of America

## Abstract

In most organisms, the heat-shock response involves increased heat-shock gene transcription. In Kinetoplastid protists, however, virtually all control of gene expression is post-transcriptional. Correspondingly, *Trypanosoma brucei* heat-shock protein 70 (HSP70) synthesis after heat shock depends on regulation of *HSP70* mRNA turnover. We here show that the *T. brucei* CCCH zinc finger protein ZC3H11 is a post-transcriptional regulator of trypanosome chaperone mRNAs. ZC3H11 is essential in bloodstream-form trypanosomes and for recovery of insect-form trypanosomes from heat shock. ZC3H11 binds to mRNAs encoding heat-shock protein homologues, with clear specificity for the subset of trypanosome chaperones that is required for protein refolding. In procyclic forms, ZC3H11 was required for stabilisation of target chaperone-encoding mRNAs after heat shock, and the *HSP70* mRNA was also decreased upon ZC3H11 depletion in bloodstream forms. Many mRNAs bound to ZC3H11 have a consensus AUU repeat motif in the 3′-untranslated region. ZC3H11 bound preferentially to AUU repeats *in vitro*, and ZC3H11 regulation of *HSP70* mRNA in bloodstream forms depended on its AUU repeat region. Tethering of ZC3H11 to a reporter mRNA increased reporter expression, showing that it is capable of actively stabilizing an mRNA. These results show that expression of trypanosome heat-shock genes is controlled by a specific RNA-protein interaction. They also show that heat-shock-induced chaperone expression in procyclic trypanosome enhances parasite survival at elevated temperatures.

## Author Summary

When organisms are placed at a temperature that is higher than normal, their proteins start to unfold. The organisms protect themselves by increasing the synthesis of “heat-shock” proteins which can re-fold other proteins when the temperature returns to normal. In trypanosomes, the degradation of mRNAs that encode heat-shock proteins is slowed down at elevated temperatures. *Trypanosoma brucei* multiplies as “bloodstream forms” in the blood of mammals, at temperatures between 37–39°C; and as “procyclic forms” in Tsetse flies, which are usually at 20–37°C but can survive at 41°C. In this paper we show that in *Trypanosoma brucei*, a protein called ZC3H11 can bind to many heat-shock-protein mRNAs. ZC3H11 is essential in bloodstream-form trypanosomes and for recovery of procyclic-form trypanosomes after heat shock. ZC3H11 binds to an AUU repeat motif which is found in parts of the target mRNAs that do not encode protein. Several heat-shock-protein RNAs were decreased when we decreased the amount of ZC3H11 in bloodstream-form trypanosomes. These and other results show that expression of the specific subset of trypanosome heat-shock proteins is controlled by the interaction of ZC3H11 with the relevant mRNAs. They also show that the heat-shock response could enhance survival of trypanosomes in over-heated Tsetse flies.

## Introduction

When living organisms are exposed to temperatures above their growth optima, they respond by increased synthesis of heat-shock proteins. In eukaryotes as diverse as animals, ciliates and plants, heat-shock protein expression is controlled by heat-shock transcription factors, whose activation enables them to bind conserved heat-shock elements in the promoters of heat-shock protein genes and activate their transcription [1], [2], [3], [4].

*Trypanosoma brucei* and related Kinetoplastid protists must also adapt to different temperatures: they multiply both in mammals, with temperatures varying from 32°C to 38°C depending on species and body location (see e.g. [5], [6], [7]), and in arthropod vectors in which the temperature variations are much greater (e.g. [8]). In Kinetoplastids, however, the regulation relies exclusively on post-transcriptional mechanisms. Transcription is polycistronic [9], [10], and individual mRNAs are produced by *trans* splicing and polyadenylation [11], [12]. The final cytoplasmic RNA level is determined by the rates of processing, transport from the nucleus, and degradation [13]. For most trypanosome mRNAs, the rate of degradation is a critical determinant of expression [14].

Two forms of *T. brucei* are routinely studied in the laboratory: the bloodstream form (found in the mammalian host, cultivated axenically *in vitro* at 37°C) and the procyclic form (found in the midgut of the Tsetse fly vector, cultivated axenically at 27°C). Upon transfer of procyclic forms to 41°C, transcription by RNA polymerase II is gradually shut down [15] and *trans* splicing is inhibited [16]. The overall level of translation also decreases, as shown by reduced *in vivo* [^35^S]-methionine labelling and the collapse of polysome profiles [17]. This is partly due to rapid mRNA degradation, as judged both by profiling of total mRNA [17] and examination of specific transcripts [18]; and it is partly due to effects on translation [17]. After heat-shock, poly(A) binding protein and several translation factors accumulate in granules [17]. Meanwhile, the mRNAs encoding HSP83 and the major cytosolic HSP70 remain stable and continue to be translated [17], [18]. Although the trypanosomes are able to recover from a 41°C heat shock lasting up to 2 hours, it is not known whether the heat-shock response is required for the recovery. Indeed, it is not known whether the trypanosome heat-shock response has any selective advantage.

*T. brucei* has five virtually identical genes encoding the major cytosolic HSP70 that are arranged in a tandem array [19] (unfortunately collapsed to one locus, Tb927.11.11330, in the genome assembly) and are constitutively co-transcribed [20], [21]. Using reporters, it was shown that sequence elements in the *HSP70* 3′-untranslated region (3′-UTR) are responsible for the stability of the mRNA after heat-shock [18], [22]. Similar observations were also made for HSP70s of the Kinetoplastids *Trypanosoma cruzi* [23] and *Leishmania infantum* [24], [25]. The multiple copies of the *HSP83* genes (encoding the major Kinetoplastid HSP90 homologue) are also in a tandem array. The 3′-UTR of *Leishmania HSP83* mRNA is important for both mRNA stability and increased translation during heat-shock [26], and it was proposed that temperature-induced changes in RNA secondary structure might play a role in regulation [27]. Post-transcriptional mechanisms are also responsible for heat-induced increases in *Leishmania HSP100* mRNA [28], [29].

The stability, localization and translation states of eukaryotic mRNAs are influenced by proteins that bind to them. For example, in mammalian cells, tristetraprolin (also called TTP, Tis11a, and Zfp36), and BRF1 and BRF2 (Butyrate response factors 1 and 2) bind to AU-rich elements with a consensus of UAUUUAUU; they recruit components of the mRNA degradation machinery, promoting mRNA decay [30]. These three proteins, together with related proteins from other Opisthokonts (together called the “Tis11 family”), possess two C_8_C_5_C_3_H zinc finger domains separated by a linker of about 10 amino acids. Immediately preceding the zinc finger domain is a six-residue conserved sequence, R/K-Y-K/R-T-E/K-L, which strongly influences the sequence specificity of RNA binding [31]. The activities of TTP and BRF proteins are regulated by phosphorylation, and are critical for control of inflammation and cell proliferation in mammals [30]. Other proteins compete for binding to the AU-rich element and promote mRNA stability [32].

*T. brucei* has forty-nine CCCH zinc finger proteins, some of which have been implicated in control of gene expression [33], [34], [35], [36], [37], [38], [39]. So far, however, none has been shown to have a destabilising function. In search of possible destabilising proteins, we looked for predicted trypanosome proteins with the Tis11 consensus. We here show that the protein with the best match, ZC3H11, indeed binds to mRNAs containing an AUU sequence element but that - in contrast to the situation in mammalian cells - the consequence is an increase in mRNA abundance. Most interestingly, ZC3H11 appears to be a master regulator of stress response mRNAs.

## Results

### ZC3H11 has a zinc finger similar to those of TTP and BRF2

**Figure 1:**
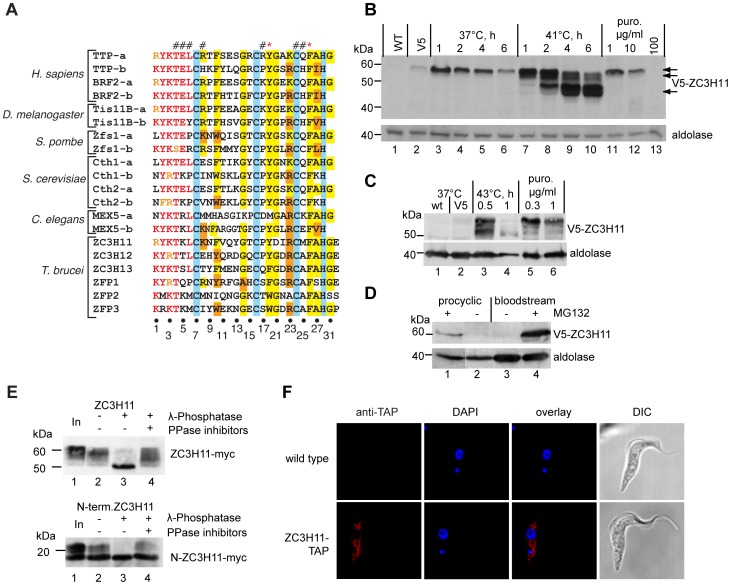
Expression of ZC3H11 is induced by stress. **A.** Alignment of ZC3H11 with members of the Tis11 family of several species. The conserved amino acid signature (marked in red) precedes the zinc finger domain (cysteines and histidine shaded in blue). Conserved residues are shaded in yellow and chemically similar ones in orange. Amino acids at the positions marked on the top with # and * are involved in RNA binding by hydrogen bonds or base stacking respectively [31]. **B.** Expression of *in situ* tagged V5-ZC3H11 in procyclic cells under different conditions. The incubation temperature is shown above along with the duration of the incubation. Treatment with puromycin was for one hour (lanes 11–13). 10^7^cells were loaded per lane; detection was with anti-V5 antibody and with anti-aldolase as loading control. **C.** Expression of *in situ* tagged V5-ZC3H11 in bloodstream-form trypanosomes under different conditions. Details are as for (B) **D.** Effect of proteasome inhibition by treatment with MG132 (10 µg/ml, 1 h) on V5-ZC3H11 expression. **E.** ZC3H11-myc is phosphorylated. Extracts from 5×10^6^ cells were incubated with lambda phosphatase, in the presence or absence of inhibitors [64]. Both the full-length protein (upper panel) and the N-terminal fragment of ZC3H11 (first 128 amino acids; lower panel) are phosphorylated. **F.** ZC3H11-TAP is localized in the cytoplasm. TAP-tagged protein was detected by immunofluorescence. DAPI - DNA stain, detecting nucleus and kinetoplast (mitochondrial DNA); DIC - differential interference contrast.

To find CCCH proteins that might be involved in post-transcriptional gene regulation in *T. brucei*, we scanned all of them for the Tis11 consensus. The best matches were in ZC3H11, ZC3H12 and ZC3H13; ZFPs 1–3 also showed some similarity (Figure 1A). ZC3H11 (locus Tb927.5.810) consists of 364 amino acids, and has a predicted molecular weight of 39.6 kDa. The zinc finger starts at residue 70, and is preceded by the Tis11 consensus RYKTKL. The *ZC3H11* gene is found in all available Kinetoplastid genomes. Most sequence identity is concentrated around the zinc finger: comparing all available proteomes, the 6mer has consensus RYKTK(L/Y/F). Some additional conserved patches are the sequence H(N/D)PY around residue 200 of *T. brucei* ZC3H11 and a serine-rich region near the C-terminus (Supplementary Figure S1).

Alignment of the ZC3H11 zinc finger with those of other Tis11 family proteins showed that some of the residues required for interaction with AU-rich elements were conserved. From a crystal structure of BRF2 (Tis11-d) with UUAUUUAUU [31], it was found that each zinc finger of BRF2 specifically binds the sequence UAUU. Using the residue numbering in Figure 1A, and numbering the 4-nt bound RNA as U(1)-A(2)-U(3)-U(4), Tyr18 intercalates between the Us in the 3rd and 4th positions (U3 and U4), and Phe26 intercalates between U1 and A2. These residues are conserved in ZC3H11. Specificity for U1 was conferred by backbone hydrogen bonds with (Asn/His)25 and Glu5; these residues are not conserved in ZC3H11, which has a basic residue at position 5, like *C. elegans* MEX5 (Figure 1A). In contrast, A2 is hydrogen-bonded by Leu6 and Arg8, which are conserved. A notable difference between ZC3H11 and other Tis11-family zinc fingers is the presence of a novel Asp residue at position 21 in place of the conserved glycine.

### Expression of ZC3H11 is increased by heat-shock

Attempts to generate a polyclonal antibody that could detect ZC3H11 in parasite lysates failed. In order to detect ZC3H11 in trypanosomes, we therefore integrated a sequence encoding an N-terminal V5-epitope tag [40] in frame with one of the *ZC3H11* open reading frames (ORFs). Since the 3′-UTR is conserved by this procedure, expression levels are expected to be approximately normal unless the tag affects protein stability. In both procyclic forms, which we normally grow at 27°C, and the bloodstream stage, grown at 37°C, the V5-ZC3H11 fusion protein was detected as an extremely faint band that migrated at about 60 kDa instead of the expected 40 kDa (Figure 1B & C, lane 2). The abundance of V5-ZC3H11 was, however, dramatically increased upon heat shock. In procyclic forms, induction of ZC3H11 was transient at 37°C (Figure 1B lanes 3–6), but stronger and more extended at 41°C; at later time points, some smaller products appeared (Figure 1B lanes 7–10). Since the longer incubations resulted in a decline in cell viability, the faster-migrating bands could indicate either proteolytic degradation or the removal of posttranslational modifications. For bloodstream forms, incubation at 43°C led to a rapid induction of V5-ZC3H11 although the cells started to die within an hour (Figure 1C lanes 3,4). Further experiments showed that in addition to elevated temperatures, mild translational stress from low concentrations of puromycin also increased V5-ZC3H11 expression in both developmental stages (Figure 1B lane 11–13 and 1C lanes 5,6). In a preliminary attempt to determine the mechanism of this expression regulation, we incubated cells with lactacystin (not shown) or MG132 (Figure 1D) to inhibit the proteasome. Indeed, the amount of ZC3H11 increased (Figure 1D). This might mean that the protein is normally rapidly degraded by the proteasome, but is stabilised upon heat shock or puromycin stress. Alternatively, proteasome inhibition could be acting as another sort of stress, with ZC3H11 protein increasing by another mechanism.

We also expressed ZC3H11-myc in cells with V5-ZC3H11. Reciprocal pull-downs revealed no evidence for dimerization (not shown).

### ZC3H11 is phosphorylated and localizes in the cytoplasm

To determine the nature of the possible post-translational modifications, we incubated cell lysates with λ-phosphatase before electrophoresis. The 60 kDa bands (Figure 1E, upper panel, lane 1) collapsed to one band at approximately 50 kDa (Figure 1E, upper panel, lane 3); this was prevented by addition of phosphatase inhibitors (Figure 1E, upper panel, lane 4), showing that the 10 kDa migration difference was indeed due to phosphorylation. Interestingly, the extra band seen after heat shock at 41°C had a similar migration (Figure 1B lanes 8–10, Figure 1C, lane 3), suggesting that it too might have been dephosphorylated. For an N-terminal fragment containing the first 128 residues of ZC3H11, extending 39 residues beyond the zinc finger, a similar pattern was observed (Figure 1E, lower panel), except that a portion appeared unmodified. The N-terminal fragment ran as several bands between 23 kDa and 17 kDa, which all collapsed to the lowest band upon phosphatase treatment. This indicates that residues in the N-terminal region can be phosphorylated.

The low abundance of the V5-*in situ* tagged protein precluded localisation by microscopy or cell fractionation. We therefore instead looked at the location of ZC3H11 bearing a tandem affinity purification (TAP) tag, inducibly expressed from a strong RNA polymerase I promoter in procyclic forms. ZC3H11-TAP was clearly excluded from the nucleus and found in the cytoplasm in somewhat granular structures (Figure 1F). The presence of an IgG-binding domain in the tag prevented us from looking for colocalisation with stress granule markers. Inducibly expressed ZC3H11 with a C -terminal myc tag gave similar results but with a much fainter signal: a rather granular cytoplasmic immunofluorescence which became marginally brighter after a one-hour heat shock (Supplementary Figure S1B).

### RNA-binding specificity of ZC3H11 *in vivo*

To find out which mRNAs were bound by ZC3H11, we inducibly expressed myc-tagged ZC3H11 in procyclic trypanosomes, precipitated the protein using anti-myc antibody, and compared bound and unbound RNAs by RNASeq. The twenty-four most strongly enriched transcripts are listed in Table 1 and the full list is in Supplementary Table S1, sheet 1. Strikingly, more than half of the strongly bound mRNAs were implicated in the stress response. Thirteen of them encoded a full set of chaperones required for protein refolding. All classes of cytosolic HSPs were represented - HSP70, HSP83 (HSP90 family), HSP100, HSP110 and HSP20. Also, mRNAs encoding putative homologues of co-chaperones were present: DnaJ (HSP40) proteins, a FKBP/TPR domain protein, stress induced protein 1 (STI1) and cyclophilin-40. Three additional bound mRNAs encoded the mitochondrial chaperone HSP60, a copper chaperone for cytochrome c, and the glutaredoxin GRX2, which protects against oxidative stress. Notably, mRNAs encoding chaperones for co-translational folding (TRiC complex) or organellar import (mitochondrial HSP70 and ER-resident BiP were not enriched. Among bound transcripts that do not encode annotated chaperones, the most notable encoded GPEET procyclin. Five bound mRNAs encoded proteins of unknown function.

**Table 1:** Possible mRNA targets of ZC3H11.

Gene ID	description	PC bind	PC HS RNAi	PC HS Seq	BS RNAi Seq	(AUU)_3_
Tb927.11.11330	Heat shock protein HSP70	4.2	0.2	1.1*	0.5	yes
Tb927.10.10980	Heat shock protein HSP83	3.9	0.2	0.7	1.7	yes
Tb927.2.5980	Heat shock protein HSP100	3.2	0.2	4.1§	1.6	yes
Tb927.10.12710	Heat shock protein HSP110	3.2	0.1	0.7	0.9	yes
Tb927.5.2940	Stress-induced protein STI1	4.1	0.1	1.0	1.1	yes
Tb927.1.3200	SGT1-like	9.5	0.3	9.0	1.0	(yes)
Tb927.9.9780	CYP40-like PPIase	3.6	0.1	1.1	1.0	no
Tb927.10.16100	FKBP-type PPIase	10.1	0.3	3.2*	0.9	yes
Tb927.2.5160	DNAj-like DNAJ2	6.1	0.1	4.0*	1.0	yes
Tb927.11.16980	DNAj-like DNAJ1	3.3	0.3	0.4	0.9	(yes)
Tb927.10.8540	DNAj-like DNAJ6	3.1	0.5	5.8	1.4	yes
Tb927.11.15480	HSP20 domain	9.6	0.3	6.3	2.0	yes
Tb927.7.4290	HSP20 domain	3.1	0.3	2.8	1.2	yes
Tb927.3.2650	Cytochrome c oxidase copper chaperone	3.6	1.0	1.3	1.4	yes
Tb927.10.6510	Mitochondrial HSP60	3.4	0.1	1.1	0.9	(yes)
Tb927.1.1770	glutaredoxin GRX2	3.2	0.6	2.6	1.9	yes
Tb927.6.510	GPEET2 procyclin	6.6	1.5	35	6.9	no
Tb927.1.3390	Hypothetical, conserved, DUF866	11.8	0.3	28.6	1.4	yes
Tb927.11.2160	Hypothetical, conserved, 1TM	9.5	0.6	4.8	1.4	(yes)
Tb927.3.5410	Hypothetical, conserved	3.8	1.3	3.6	1.7	no
Tb927.9.4960	Hypothetical, conserved	3.8	1.2	7.4	1.8	yes
Tb927.10.780	Hypothetical, conserved, Ring Zn finger	5.3	0.8	11.8§	1.2	no

The Table includes all mRNAs that were at least 3-fold enriched in the ZC3H11 bound fraction, with more than 40 reads in the bound fraction and more than10 reads per million in the unbound fraction. The full lists are in Supplementary Table S1. The genes are grouped according to predicted protein function. All RNASeq results were normalised to reads per million per kilobase (rpkm) before the ratios were calculated. PC bind: Ratio of eluate to flow-through in the binding experiment. “Description” is the functional classification in TriTrypDB, with some additions and modifications. PC HS Seq: RNA rpm for procyclics heated to 41°C for 1 h, relative to procyclics kept at 27°C. PC HS RNAi: RNA read counts per million in procyclics with *ZC3H11* RNAi, heated to 41°C for 1 h, divided by the read counts in wild-type procyclics heated to 41°C;

* Northern blot result between 1× and 1.5× (Supplementary Figure S5);

§ Northern or published microarray result 5–6× [17]. BS RNAi Seq: effect of RNAi targeting *ZC3H11* in bloodstream forms, relative to wild-type. “(AUU)_3_”: “yes” indicates the presence of (at least) the 12mer AUUAUUAUUAUU in the 3′-UTR, (yes) is a perfectly-matching 11mer. PPIase = peptidyl prolyl cis-trans isomerase; TM = trans-membrane domain, DUF866 = domain of unknown function. For multi-copy genes only one member is shown. They are *HSP83* (Tb917.10.10890-10980), *HSP60* (Tb927.10.6400 and 6510), and *HSP70*.

**Figure 2:**
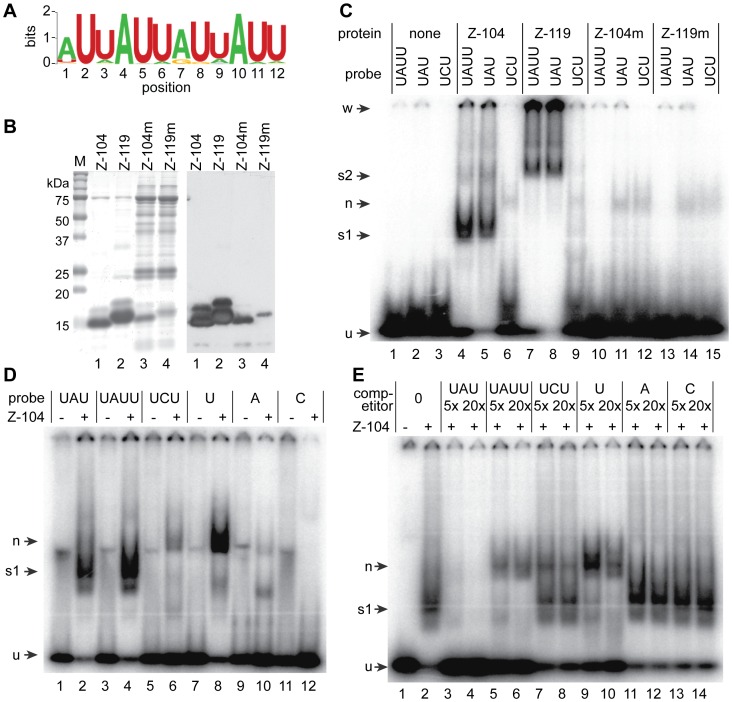
The ZC3H11 zinc finger can bind to AUU repeats. **A.** The 12mer motif enriched in the 3′-UTRs of transcripts that were enriched in the ZC3H11 bound fraction, as measured by RNASeq. The motif was identified using MotifSampler. **B.** Purified His-tagged ZC3H11 fragment preparations were separated by SDS-PAGE and either stained with Coomassie (left) or blotted and detected using anti-His antisera (right). Proteins were (1) His-tagged N-terminal 104mer; (2) His-tagged N-terminal 119mer; (3) His-tagged N-terminal 104mer with C70S mutation; (4) His-tagged N-terminal 119mer with C70S mutation. **C.** The His-tagged ZC3H11 fragments (100 pmol total protein) were incubated with 1 pmol radioactively labelled probe in the presence of 10 mg/ml heparin, then separated by native PAGE. The phosphorimager output is shown. Radioactive RNA probes were: UAU: U(UAU)_7_U; UAUU: (UAUU)_5_UAU; UCU: U(UCU)_7_U. Arrows indicate unbound probe (u), a small UAU or UAUU-specific complex (s1); a larger UAU or UAUU-specific complex (s2); a non-specific complex (n); and aggregates in the well (w). **D.** The His-tagged N-terminal 104mer of ZC3H11 (100 pmol total protein, Z-104) was incubated with 1 pmol radioactively labelled probe, in the presence of 5 µg/ml heparin, then separated by native PAGE. The phosphorimager output is shown. Radioactive RNA probes were as follows: UAU: U(UAU)_7_U; UAUU: (UAUU)_5_UAU; UCU: U(UCU)_7_U; U: (U)_23_; C: (C)_23_; A: (A)_23_. Arrows indicate unbound probe (u), a UAU or UAUU-specific complex (s1) and a different UCU or poly(U)-specific complex (n). **E.** The His-tagged N-terminal ZC3H11 104mer (100 pmol total protein) was incubated with 1 pmol radioactively labelled U(UAU)_7_U in the presence of 5 µg/ml heparin and competing oligonucleotides in 5 or 20-fold excess. Remaining details are as in (C). Addition of 10 mg/ml heparin did not affect the result (Supplementary Figure S2E).

We next analysed the 3′-UTRs of all bound transcripts for enriched motifs. We found a striking enrichment of an (AUU)n repeat motif (Figure 2A, supplementary Table S1, sheet 2). Of the 22 most strongly bound mRNAs, 14 contained perfect (AUU)_4_ repeats and four more had a repeat of 11 nt (Table 1): they included all but one (*CYP40*) of the chaperone mRNAs. A scan of the whole genome revealed 325 genes with a good match to the 12mer (AUU)_4_ sequence in their predicted 3′-UTR, of which only 44 were at least two-fold enriched in the ZC3H11-bound fraction. Although the 3′-UTRs used in the analysis may, in some cases, be incorrect, this result shows that the presence of an (AUU) repeat alone is not sufficient to give ZC3H11 binding.

### RNA-binding specificity of ZC3H11 *in vitro*

The putative AUU repeat binding motif is interesting because the sequence bound by a single Tis-11 CCCH domain is UAUU [31]. To investigate the RNA-binding specificity of ZC3H11 in more detail, we expressed a variety of different fusion proteins in *E. coli* and purified them. The only proteins that could be obtained in reasonable quantity and purity were two N-terminal fragments of 104 and 119 residues (Figure 2B, lanes 1 & 2). Both contain the zinc finger but the 119mer also includes additional conserved residues (Supplementary Figure S1A). Although the proteins formed single bands on denaturing gels (Supplementary Figure S2A), on native gels, the pattern was very smeared and some protein remained in the well (Supplementary Figure S2B). This suggested that despite initial solubility, the proteins were not fully folded and some aggregation was occurring. As controls, we expressed the same protein fragments with a C70S mutation in the zinc finger. These were very poorly expressed and as a consequence, the purified samples were heavily contaminated (Figure 2B, lanes 3 and 4).

To test for RNA binding, we incubated the proteins with various radioactively-labelled oligo-ribonucleotides and examined migration in non-denaturing polyacrylamide gels. The 104 residue protein (ZC3H11-104), interacted with both (UAUU)_5_UAU (classical ARE) and U(UAU)_7_U to give a clear band (Figure 2C, lanes 4 & 5, arrow s1). U(UCU)_7_U gave a very faint band of slower mobility (Figure 2C, lane 6, arrow n) which was also detected for both U(UAU)_7_U and U(UCU)_7_U using the zinc finger mutants (Figure 2C, lanes 11, 12, 14,15). This band most likely represents binding of the probe by an *E. coli* contaminant, although zinc-finger-independent binding by ZC3H11 is also possible. Using the 119-residue protein, ZC3H11-119, a slower-mobility band was obtained using (UAUU)_5_UAU and U(UAU)_7_U (Figure 2C, lanes 7 & 8, band s2), suggesting binding of additional copies of the protein: perhaps the extra 15 amino acids mediate protein-protein interactions. In addition, there was strong accumulation of radioactivity in the well (w). This could be explained if the zinc finger were properly folded, but the remainder of the polypeptide were unfolded and formed aggregates. Reducing the probe length to 14 residues did not affect the apparent aggregation (not shown).

To investigate the binding in more detail, we used more probes. Results for ZC3H11-104 are shown in Figure 2D and 2E. The interactions with U(UAU)_7_U and (UAUU)_5_UAU were confirmed (Figure 2D, lanes 2 & 4, arrow s1), and the faint band using U(UCU)_7_U (Figure 2D, lane 6, arrow n) was much stronger using U_23_ (Figure 2D, lane 8, arrow n). A very faint shift was seen with A_23_, but none with C_23_ (Figure 2D, lanes 10 & 12). To further assess specificity, ZC3H11-104 was incubated with the labelled U(UAU)_7_U probe in the presence of unlabelled competitors. U(UAU)_7_U competed effectively, most of the probe now remaining unbound (Figure 2E, lanes 3 and 4). In contrast, addition of cold (UAUU)_5_UAU (Figure 2E, lanes 5 and 6) or of U_23_ shifted the radioactivity to the non-specific band (Figure 2E, lanes 9 and 10, band n). U(UCU)_7_U showed partial competition (Figure 2E, lanes 7 and 8) whole A_23_ and C_23_ could not compete at all. Results for ZC3H11-119 were similar except that as before, the specific complex showed slower migration and radioactivity accumulated in the well (Supplementary Figure S2C–E). The strong shift to the apparently less specific band (“n”) in the competition assays was not inhibited by heparin (Supplementary Figure S2E).

We also attempted to assess the binding affinities of ZC3H11-119 to limiting amounts of U(UAU)_7_U, (UAUU)_5_UAU and U_23_ probes. The only probe that bound at all under these conditions was U(UAU)_7_U, but the results could not be interpreted quantitatively because at most protein concentrations, the only bound radioactivity was stuck in the well (Supplementary Figure S2F).

We concluded that the zinc finger of ZC3H11 binds preferentially to (UAU) repeats, but is also able to bind to the classical ARE.

### ZC3H11 RNAi decreased HSP70 mRNA in bloodstream-form trypanosomes

**Figure 3:**
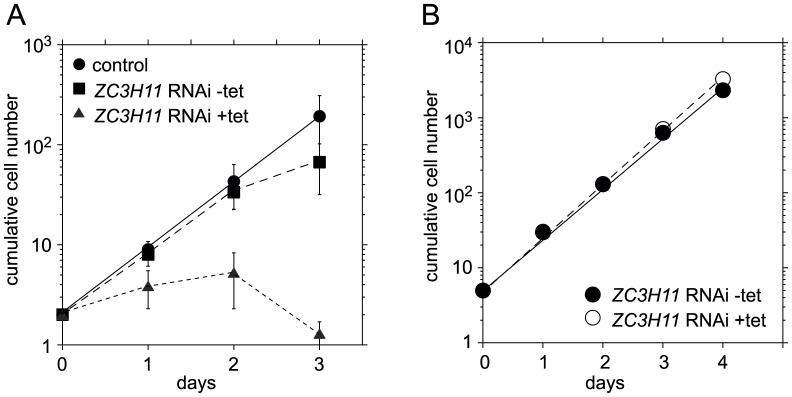
ZC3H11 depletion kills bloodstream forms but not procyclics. **A.** Effect of *ZC3H11* RNAi on growth of bloodstream forms. Tetracycline was added at day 0, and cells were diluted as required. Cumulative growth curves are shown. Results are for four independent cell lines, immediately after isolation. Results from two experiments are included. In experiment one, three lines were tested over three days of tetracycline treatment. In experiment 2, the same lines plus another were examined for two days with tetracycline. The control is from experiment 1, and shows pooled results for 11 trypanosome lines with RNAi targeting a non-essential gene, growth without tetracycline for three days. Results are shown as arithmetic mean ± standard deviation. **B.** Effect of *ZC3H11* RNAi on growth of procyclic forms, cumulative growth curve. Results are shown from triplicate measurements from a line with stem-loop RNAi, as arithmetic mean ± standard deviation. The error bars are not visible because they are smaller than the symbols.

We examined the effect of ZC3H11 depletion by RNA interference (RNAi). Stable cell lines inducibly expressing a double stranded RNAi fragment were created in bloodstream- and procyclic-form trypanosomes. In bloodstream forms, depletion of ZC3H11 was lethal (Figure 3A), while no effect was observed in procyclic cells (Figure 3B). A similar result was obtained in a published high-throughput RNAi screen [41].

To further investigate the reason why ZC3H11 was essential in bloodstream-form trypanosomes, the transcriptome of ZC3H11-depleted cells was compared with that of wild-type cells, initially using an oligonucleotide microarray (not shown) and later, using poly(A)+ RNA, by high-throughput cDNA sequencing (RNA-Seq) (Supplementary Table S1, sheet 3 and Supplementary Figure S3A,B). The RNA from ZC3H11-depleted cells was taken 24 h after induction of RNAi, before a growth defect was evident, and with no drug treatment apart from tetracycline, which is known not to affect the transcriptome at the level used [14]. We compared the RNASeq results with a previous dataset for poly(A)+ RNA from wild-type cells. 452 transcripts were at least 2-fold increased after ZC3H11 depletion (Supplementary Table S1, sheet 3). The increased transcripts were significantly enriched in the categories of protein kinases and phosphatases, and also RNA-binding proteins, but have not yet been examined further. There was no correlation between the effects of *ZC3H11* RNAi in bloodstream forms and enrichment in the ZC3H11-bound fraction, suggesting that many of the effects seen were secondary. Bound RNAs that increased included GPEET procyclin, but since procyclin-associated mRNAs, which are in the same transcription unit, also increased, an increase in procyclin locus transcription is possible. Other increased ZC3H11-bound mRNAs included those encoding the putative RNA-binding protein RBP5 and a few proteins of unknown function.

**Figure 4:**
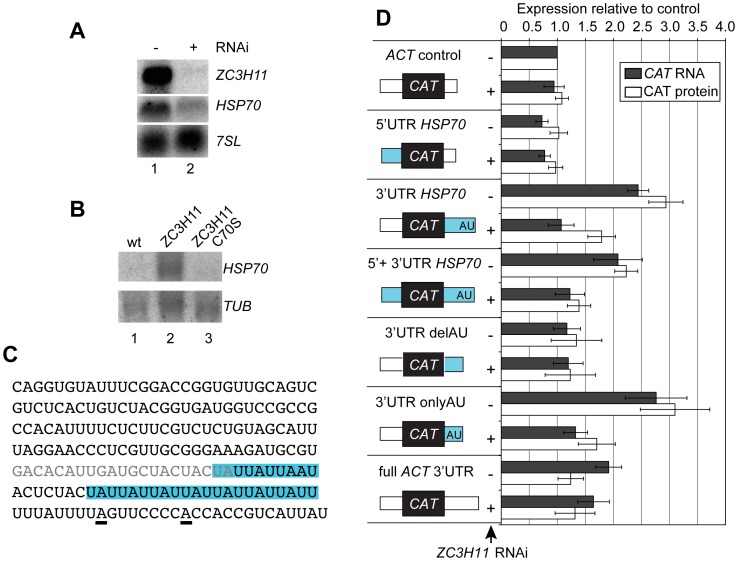
ZC3H11 depletion decreases the *HSP70* mRNA level and this effect requires the AU-rich sequence in the 3′-UTR. **A.** Effect of *ZC3H11* depletion on *HSP70* mRNA levels in bloodstream forms. *HSP70* and *ZC3H11* RNA were detected by Northern blotting after one day of RNAi induction, with the *7SL* RNA as loading control. **B.** *HSP70* RNA co-precipitates with myc-tagged ZC3H11. Extracts from trypanosomes expressing no myc-tagged protein (wt), ZC3H11-myc, or ZC3H11-myc with the first cysteine of the zinc finger mutated to serine (C70S), were immunoprecipitated with anti-myc antibody. RNA was purified from the precipitates and analysed by Northern blotting. The blot was probed for *HSP70*, then reprobed to detect tubulin RNA in order to assess non-specific binding. **C.** Sequence of the *HSP70* 3′-UTR amplified from the genome. The underlined A's are alternative polyadenylation sites [22]. The blue shadowed regions are the AUU repeats. The grey type indicates the primer used to amplify the 5′ and 3′ regions of the 3′-UTR; this sequence is present in both constructs. **D.** The AUU repeat region is necessary and sufficient for the response to ZC3H11. Stable transgenic bloodstream-form trypanosome cell lines with inducible *ZC3H11* RNAi and constitutively expressed *CAT* reporter constructs that integrated into the tubulin locus (pol II transcription) were generated. In the controls, the *CAT* ORF is flanked by a *EP* 5′-UTR and either a truncated actin (*ACT*) 3′-UTR or the full-length *ACT* 3′-UTR (at the bottom). The other constructs have full length or truncated *HSP70* UTRs (blue) as indicated. Relative CAT expression levels, with or without RNAi induction (1 day), were determined by Northern blot and CAT activity, and expressed as arithmetic mean ± standard deviation of at least 3 measurements.

RNASeq revealed 72 genes with at least 2-fold decreased mRNA expression after ZC3H11 RNAi. The strong enrichment for genes encoding ribosomal proteins (P = 5×10^−15^) and translation factors (P = 0.05) suggests that some of the decreases could be indirect effects, secondary to the onset of growth arrest. Looking at ZC3H11-bound mRNAs (Table 1), one encoding an FKBP-like petidyl-prolyl cis-trans isomerase was not decreased according to the RNASeq, but was decreased by Northern blotting (0.2×, Supplementary Figure 3C) and microarray (0.4×, not shown). *HSP70* mRNA levels were reproducibly halved by RNASeq, microarray (not shown) and Northern blotting (Figure 4A and Supplementary Figure 4B). We therefore decided to investigate *HSP70* regulation by ZC3H11.

### ZC3H11 regulates HSP70 via an (AUU)n repeat in the 3′-UTR

To confirm binding of *HSP70* mRNA to ZC3H11, we immunoprecipitated ZC3H11-myc from procyclic trypanosome extracts and subjected the resulting RNA to Northern blotting (Figure 4B). As controls, we used cells that expressed no myc-tagged protein, or cells expressing a myc-tagged version of ZC3H11 with the C70S mutation. Since the immunoprecipitation is a lengthy procedure, some degradation of the mRNA occurred, but nevertheless, a band of *HSP70* mRNA was visible in the preparation from cells expressing ZC3H11-myc, whereas no *HSP70* mRNA was detected in the control pull-downs. As a further control for non-specific RNA sticking to the beads we looked for the highly abundant tubulin mRNA. As expected, some of this mRNA was found in all lanes, but with no specificity for pull-down by ZC3H11-myc (Figure 4B). The C70S mutant protein was rather poorly expressed relative to the wild-type (not shown), so this experiment by itself allows no conclusions regarding a requirement for the C70 residue of the zinc finger in RNA binding.

To find out whether the effect on *HSP70* mRNA abundance in bloodstream-form trypanosomes was caused by increased instability, we inhibited transcription and measured the amount of *HSP70* mRNA left after 15 and 30 min. In five independent measurements, the *HSP70* mRNA half life was 23±7 min (mean ± standard deviation). The RNAi cell line yielded values of 21±9 min in the absence of tetracycline, and 15±6 min after one day of RNAi induction. *ZC3H11* RNAi decreased the half-life of *HSP70* mRNA in every experiment, suggesting that ZC3H11 stabilises *HSP70* mRNA.

It was already known that trypanosome *HSP70* mRNA abundance is regulated by the 3′-UTR [17], [18], [22]. To define the region that was targeted by ZC3H11, we generated bloodstream-form cell lines that had inducible RNAi against *ZC3H11*, and also constitutively expressed chloramphenicol acetyltransferase (CAT) reporter constructs flanked by different UTRs (Figure 4C, 4D and Supplementary Figure S4A). The constructs were integrated into the tubulin locus and expressed by read-through transcription by RNA polymerase II. Reporter protein expression was measured by the CAT assay; *CAT* RNA levels and correct mRNA processing were assessed by Northern blotting (Figure 4D and Supplementary Figure S4, B & C).

The parental construct expressed an mRNA with the 5′-UTR from the *EP* mRNA, and a truncated actin 3′-UTR (Figure 4D, control). Introducing the *HSP70* 5′-UTR caused no significant change in expression levels compared to the parental constructs (Figure 4D, *HSP70* 5′-UTR). In contrast, when the *HSP70* 3′-UTR was included, either by itself or together with the *HSP70* 5′-UTR, the steady state levels of CAT mRNA and protein were approximately twice the control. Induction of RNAi against ZC3H11 reduced this expression to the level of the control construct. These results show that the *HSP70* 3′-UTR was sufficient for ZC3H11-mediated regulation. Since all constructs were transcribed from the same locus, the mechanism must be post-transcriptional. We attempted to compare the half-lives of the *CAT-HSP70* reporter mRNAs but the low amounts present after *ZC3H11* RNAi prevented accurate quantitation.

We wanted to see whether the AU-rich sequence was required for ZC3H11-mediated mRNA stabilisation. A reporter with just the 5′ part of the *HSP70* 3′-UTR, which lacks the AU sequence element, was expressed at levels similar to the control, and showed no response to *ZC3H11* RNAi (Figure 4D, delAU). In contrast, a construct containing only the 3′ part, with mainly just the AU element, behaved like the construct with the complete 3′-UTR. We concluded that the part of *HSP70* 3′-UTR that contains (AUU) repeats is necessary and sufficient for regulation by ZC3H11 in bloodstream forms.

Finally, we inserted (TAT)_6_ either at the beginning, or at the end, of the actin 3′-UTR in the reporter plasmid. The insertion after the coding region and before the actin 3′-UTR had no effect on *CAT* RNA or protein (Supplementary Figure S4D). An insertion just before the usual poly(A) site resulted in a two-fold increase in both RNA and protein, but the effect was independent of the 18mer orientation and was not affected by ZC3H11 RNAi (Supplementary Figure S4D). This confirms our impression that in order to respond to ZC3H11, the AU-rich sequence requires a particular context. Other regulatory elements behave similarly in trypanosomes: for example, *EP* mRNA degradation in bloodstream-form trypanosomes is regulated by a 26mer [42], [43], but the 26mer alone does not work if placed at the start of the actin 3′-UTR [42].

### ZC3H11 is required for the procyclic-form heat-shock response

**Figure 5:**
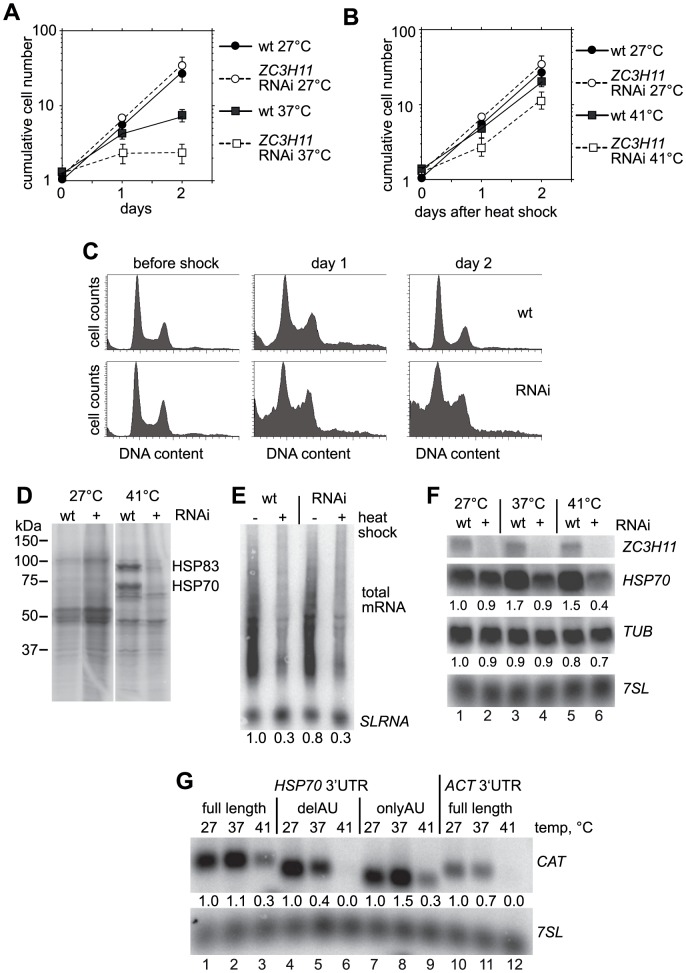
ZC3H11 is required for recovery of procyclic trypanosomes from heat shock. **A.** Effects of incubating procyclic cells at 37°C, with or without *ZC3H11* RNAi. Cells were diluted to 1×10^6^/ml as required to avoid densities above 5×10^6^/ml. Results are for three experiments, expressed as arithmetic mean ± standard deviation. If no error bars are visible, they were smaller than the symbols. **B.** Recovery of procyclic cells with or without *ZC3H11* RNAi from a one-hour heat shock at 41°C. On day 0, cells were incubated at 41°C for one hour then returned to 27°C. They were diluted on subsequent days as required. Cells with no heat shock served as controls. Results are for three experiments, expressed as arithmetic mean ± standard deviation. **C.** Analysis of DNA content of procyclic trypanosomes recovering from heat shock, with and without *ZC3H11* RNAi by FACS. The growth curve for these particular cells was included in those used to make the graph B. **D.** Effect of *ZC3H11* RNAi on heat-shock protein synthesis in procyclic forms. Cells were shocked at 41°C for one hour, then pulsed with [^35^S]-methionine. Labelled proteins were separated by SDS-PAGE and detected by autoradiography. **E.** Effect of heat shock on total mRNA abundance. RNA was prepared and analysed by Northern blotting after a one-hour heat shock at 41°C. The total mRNA was detected by hybridisation with the spliced leader (present at the 5′-end of each mRNA). The numbers below show the total signal from spliced mRNA (without the *SLRNA* itself), normalized to ribosomal RNA (measured by methylene blue staining of the blot). **F.** *ZC3H11* RNAi abolishes the specific stabilization of *HSP70* mRNA upon heat shock. Northern blots are shown, as in (E) but also including a one-hour incubation at 37°C. The quantitation, normalised to the *7SL* RNA signal, is the average of 2 independent experiments. **G.** The UAU-rich region of the *HSP70* 3′-UTR is able to stablise a reporter mRNA after heat shock. Procyclic trypanosomes expressing *CAT* reporters with segments of the *HSP70* 3′-UTR were incubated at 41°C for one hour, then RNA was prepared and analysed by Northern blotting. Constructs contained either full-length *HSP70* 3′-UTR or the fragments “delAU” or “onlyAU” (Figure 4) and the reporter with full-length actin 3′-UTR served as control. For each reporter, *CAT* RNA levels were quantified relative to the non-heat shock level and normalized using the *7SL* signal.

Since chaperones were strongly enriched among the possible ZC3H11 targets, we investigated ZC3H11 function in procyclic trypanosomes incubated above their normal culture temperature of 27°C. At 37°C, wild-type cells stopped multiplying after 3–4 days, while cells with RNAi showed a slower cell number increase and were already starting to die after 2–3 days (Figure 5A). Since the latter result suggested that ZC3H11 was important in survival at elevated temperatures, we tested published heat-shock conditions. After a one-hour incubation of our normal procyclic forms at 41°C, motility was strongly reduced, but, as previously observed [17], the cells recovered rapidly after being returned to 27°C (Figure 5B, WT). If the cells were depleted of ZC3H11, however, recovery was severely impaired (Figure 5B, RNAi). In another cell line, containing only one, V5-tagged copy of ZC3H11, recovery kinetics were intermediate between the RNAi and wild-type (not shown). This suggests that V5-ZC3H11 is functional, but the presence of only a single copy of ZC3H11 causes haplo-insufficiency.

To look at the effect of the heat shock on cell cycle progression in more detail, we analysed cell shape and DNA content by FACS (Figure 5C). Normal cells before shock had identical patterns with a G1 peak of 1× diploid DNA content, a smaller G2/M peak with 2× diploid DNA content, and cells in S-phase in between. One day after the heat shock, both populations showed relatively more G2/M cells, an accumulation of multinucleate cells with abnormally high DNA content, and some dead cells with less than 1× DNA content. The wild-type population had returned to normal by day 2, but for the population with *ZC3H11* RNAi, dead cells and cells with abnormally high DNA content persisted and the G1/G2 ratio had not recovered (Figure 5C).

As previously described [17], a one-hour 41°C heat shock reproducibly decreased *de novo* synthesis of many proteins, as judged by [^35^S]-methionine labelling (Figure 5D); among those spared were two migrating at about 90 kDa and 70 kDa, which are probably HSP83 and HSP70. This result was extremely similar to that previously seen for insect-stage *Leishmania* [44], [45]. Transcription initiation is shut down in trypanosomes after heat shock [15] and by preparing RNA, then analysing the amount of mRNA by Northern blotting with a spliced leader probe, we found that the global mRNA level was decreased after the 1 h-heat shock whether or not *ZC3H11* RNAi had been induced (Figure 5E). The mRNA encoding alpha tubulin decreased by 20–30% and mRNA encoding glycerol-3-phosphate dehydrogenase by 50% (Supplementary Figure S5). As expected, in heat-shocked cells without RNAi *HSP70* mRNA persisted (Figure 5F, lanes 1 & 2). In contrast, after ZC3H11 RNAi, stabilisation of *HSP70* mRNA was no longer seen (Figure 5F, lanes 5 & 6). Similar results were observed for *HSP83*, *HSP110*, *FKBP*, and the mRNA encoding the HSP40/DnaJ-like protein J2; moreover, *HSP100* mRNA was induced by heat shock in wild-type cells but not induced after *ZC3H11* RNAi (Supplementary Figure S5). Cultivation of the parasites at 37°C for 1 h caused a 70% increase in *HSP70* mRNA which was prevented by *ZC3H11* RNAi (Figure 5F, lanes 3 & 4).

We transfected procyclic forms with the *CAT* reporters containing the full *HSP70* 3′-UTR, the *HSP70* 3′-UTR fragments or the actin 3′-UTR (Supplementary Figure S4A) and subjected the parasites to heat shock. This revealed that the AU-rich segment from the distal portion of the *HSP70* 3′-UTR was sufficient to confer persistence of the reporter mRNA in heat shock conditions (Figure 5G, lanes 7–9) whereas the mRNA with the 5′ portion (Figure 5G, lanes 4–6) behaved similarly to the actin control (Figure 5G, lanes 10–12).

We concluded that ZC3H11 is required for the heat-shock response of procyclic trypanosomes, and that the heat-shock response is required for recovery of the parasites from incubation at 41°C.

### ZC3H11 is required for the retention of target mRNAs after heat shock

A previous microarray analysis had identified mRNAs that escape degradation after heat shock of procyclic forms [17]. We repeated this analysis by RNASeq, comparing the transcriptomes of procyclic trypanosomes after one hour at 41°C with those of parasites that remained at 27°C. A large number of mRNAs was affected (Supplementary Table S1, sheet 4 and Supplementary Figure S3D). In theory, the 41°C RNASeq data should be normalised to allow for the fact that the total amount of mRNA is four-fold diminished by heat shock (Figure 5E), which means that the read count ratio (heat shock/no heat shock) should be divided by four. In practice, however, the un-normalised RNASeq results agreed better with those from Northern blots (Table 1). We do not understand why this is the case. Of the 178 loci that showed at least 2-fold more expression in the published heat shock microarray, 88 were confirmed as at least 2-fold increased in the RNASeq analysis; examples are listed in Table 2. Intriguingly, the increased transcripts were significantly enriched for the class encoding RNA-binding proteins (P = 0.007). Several chaperones were actually decreased after heat shock, but these were preferentially those involved in vesicular transport (Supplementary Table S1, sheet 4). Some of the mRNAs that increase after heat shock are also normally preferentially expressed in bloodstream forms (Table 2). Overall, there was no correlation between mRNA changes after heat shock and binding to ZC3H11 (Supplementary Table S1, sheet 4), indicating that for most mRNAs, other regulatory mechanisms are involved in stabilisation after heat shock.

**Table 2:** Genes with increased mRNA after heat shock of procyclic forms.

Gene ID	Description	Seq	MA	BS/PC
Chaperones				
Tb927.2.5980	HSP100	4.1	6.3	0.4
Tb927.6.3120	DNAj-like	6.7	7.9	4.9
RNA-binding domain proteins				
Tb927.8.2780	RBP10	2.2	3.4	4.2
Tb927.6.3480	DRBD5	10.4	4.6	4.2
Tb927.2.3880	RNA-binding protein HNRNPH	19.0	4.3	2.6
Tb927.10.5150	ZC3H31	5.2	2.6	2.1
Tb927.10.15870	RNA binding protein, putative, SWAP domain	2.8	3.1	1.3
Tb927.11.8470	ZC3H45	13.4	5.0	0.9
Tb927.7.5380	RNA-binding protein, putative, RBD	11.2	7.1	0.8
Tb927.8.6650	DRBD12	4.3	3.5	0.4
Tb927.10.15610	zinc finger protein, putative	5.7	4.0	1.3
Signalling and cell cycle				
Tb927.9.6090	PTP1-interacting protein, 39 kDa PIP39	8.2	2.2	1.8
Tb927.11.5860	protein kinase, putative	4.4	5.0	1.0
Tb927.10.15690	anaphase promoting complex, subunit 10	2.9	2.4	1.2
Tb927.6.5020	cyclin 7 (CYC7)	16.7	4.8	0.8
Tb927.8.6340	cyclin 10 (CYC10)	16.5	4.0	1.6
Tb927.2.3720	ubiquitin-conjugating enzyme, putative	3.8	4.0	1.6
Cell surface and metabolism				
Tb927.11.1470	65 kDa invariant surface glycoprotein (ISG65)	5.3	7.1	5.0
Tb927.2.6000	GPI phospholipase C	2.5	3.1	13.8
Tb927.6.2830	GTPase activating protein, conserved	3.1	5.0	2.1
Tb927.4.2450	thioredoxin, putative	4.1	3.1	1.5
Tb927.9.12690	prenyl protein specific carboxyl methyltransferase	7.0	2.2	1.0
Tb927.11.1350	calcium uniporter protein, mitochondrial	6.1	4.5	2.4
Tb927.1.4830	Phospholipase A1	8.0	3.5	1.0
Tb927.10.7090	alternative oxidase (AOX)	13.6	4.4	5.0
Tb927.10.9760	alternative oxidase	2.1	2.1	1.1
Tb927.3.4500	fumarate hydratase, class I (FHc)	11.9	2.5	0.2
Tb927.10.790	vesicle-associated membrane protein	7.1	2.29	1.7

The list shows mRNAs with predicted functions that were found to be increased by RNASeq (Seq) and in a previous microarray analysis (MA); 54 additional ORFs encoding proteins of unknown function were also increased in both analyses. BS/PC shows the ratio of mRNA in bloodstream forms relative to procyclic forms according to Siegel et al [69]. The full list is in Supplementary Table S1, sheet 4. The raw microarray data include values for duplicate spots of each gene; in this table the higher value for regulation has been quoted.

To find mRNAs that were dependent on ZC3H11 after heat shock, we compared the transcriptomes of wild-type heat-shocked parasites with those of heat-shocked parasites with *ZC3H11* RNAi. 27% of mRNAs were at least 2-fold less abundant in the RNAi cells; less than 1% were increased. Although there was no transcriptome-wide correlation between ZC3H11 binding and the RNAi effect (Supplementary Table S1, sheet 5), every single one of the ZC3H11-bound heat-shock chaperone mRNAs was decreased in the RNAi cells (Table 1): the enrichment of chaperones in the subset that was both ZC3H11-bound and reduced in heat-shock was highly significant (P = 1.6×10^−13^) (Supplementary Table S1, sheet 7). The RNASeq results therefore showed that ZC3H11 is required for the retention of mRNAs encoding refolding chaperones after heat shock. The decreases in other mRNAs in the RNAi cells may be secondary to the loss of chaperones or other proteins encoded by ZC3H11-bound mRNAs.

### ZC3H11 can actively stabilise a bound mRNA

**Figure 6:**
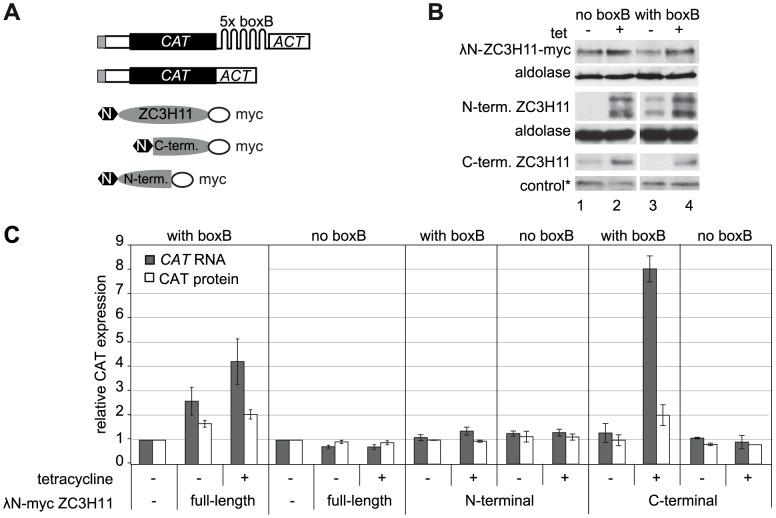
Tethering of ZC3H11 increases the abundance of a reporter mRNA. **A.** Constructs used for the tethering experiments. Above: permanent cell lines were made with constitutive production of a *CAT* reporter mRNA, with or without five “boxB” recognition sites in the 3′-UTRs. Below: additional cell lines were generated with tetracycline-inducible expression of ZC3H11 (full length or fragments) with an N-terminal lambda-N peptide and C-terminal myc tag. **B.** Expression of ZC3H11 fusion proteins. Western blotting, using anti-myc, with anti-aldolase or a cross-reacting non-specific band (*) as loading control (1×10^7^ cells per lane). **C.** Expression of CAT in cells with or without inducible expression of lambda-N-ZC3H11-myc, or the fragments. RNA and protein extracts were prepared from cells with or without tetracycline treatment to induce fusion protein expression (100 ng/ml, 24 h). RNA was measured by Northern blotting and CAT enzyme activity was assayed. Results are arithmetic mean ± standard deviation of at least 3 measurements. The increase of CAT with lambda-N-ZC3H11-myc without tetracycline addition is probably due to leaky expression (see B).

There are two basic ways in which an RNA-binding protein can stabilise an mRNA. One possibility is that it has a direct stabilising function, for example by binding to other proteins such as translation factors or poly(A)-binding protein. The other option is that it prevents binding of other, degradation-promoting, proteins to the recognition sequence. To distinguish between these possibilities, we artificially forced ZC3H11 to bind to a *CAT* reporter RNA that would not normally bind ZC3H11. To do this we took advantage of the very strong and specific binding of the phage lambdaN protein to an RNA sequence called boxB. Briefly, we expressed a ZC3H11 protein fused to the peptide with binding activity (λN-ZC3H11) (Figure 6A) together with a reporter RNA. The *CAT* reporter RNA was followed by five copies of the boxB sequence, then the actin 3′-UTR (Figure 6A), and the same reporter mRNA without boxB served as a control. The effect of induction of the λN-ZC3H11 fusion protein (Figure 6A, B) on the *CAT* reporter mRNA and the protein level were examined. λN-ZC3H11 expression resulted in a 4-fold increase in reporter RNA, and a 2-fold increase in CAT protein (Figure 6C). This effect required ZC3H11 binding, since the reporter lacking the boxB element did not react to ZC3H11 expression (Figure 6C). We do not understand the difference between the protein and RNA effects; one possibility is that the tethered ZC3H11 has a negative influence on translation. We concluded that ZC3H11 is able to stabilise transcripts actively, rather than by inhibiting the action of a destabilising protein. Tethering the C-terminal part of ZC3H11 alone, without the zinc finger domain, also increased the reporter RNA and protein, while the N-terminal zinc-finger fragment did not (Figure 6C). Together with our previous results, this suggests that the N-terminal zinc finger is required for RNA binding, while the effector domain for stabilisation lies towards the C-terminus.

## Discussion

In this paper we show that ZC3H11 is an effector of a post-transcriptional regulon for protein folding and protection against heat shock. ZC3H11 is essential for survival of the bloodstream stage of *T. brucei* and is required for heat-tolerance of procyclic forms. It is required to maintain the normal abundance of the major *HSP70* mRNA in bloodstream forms, and selectively binds to mRNAs that encode chaperones that are involved in stress responses. Many ZC3H11-bound mRNAs contain AUU repeats in their 3′-UTRs.

### Sequence specificity of the ZC3H11-RNA interaction

Experiments with recombinant ZC3H11 fragments showed that, as expected, the zinc finger was able to interact with RNA, with little discrimination between AUU and AUUU repeats. With an RNA:protein ratio of 1∶100 (Figure 2D), interaction with poly(U) was observed, but no binding was seen with ten-fold less RNA probe (not shown) and poly(U) competed extremely poorly with AUU repeats (Figure 2E). This suggested that binding to poly(U) was weak. As for MEX5 [46], no interaction was observed with poly(C) or poly(A).

The ability of ZC3H11 to discriminate against poly(U) was unexpected. In BRF2, the glutamate of the (R/K)YKTEL motif is important for the specific interaction with the adenine base [31]. In ZC3H11, lysine is found at this position - as in *Caenorhabditis elegans* MEX5. MEX5 shows little discrimination between a classical 34mer ARE (a mixture of (AUU) and (AUUU) repeats) and U_30_; mutating the lysine to glutamate in both of the MEX5 zinc fingers to generate a more TTP/BRF2-like 6mer, (N/K)YKTEL, eliminated poly(U) binding [46].

The ability of Tis11 proteins to distinguish between different 3′-UTRs is dependent on the presence of two zinc finger motifs, resulting in a minimum 8-nt binding site of two UAUU motifs. The spacing between the A residues is defined by the distance between the two zinc fingers [31]. Monomeric ZC3H11, in contrast, could recognise only UAUU so would have almost no ability to discriminate between mRNAs. The fact that the RNA pull-down yielded a highly specific motif of AUU repeats indicates that ZC3H11 must, in fact, bind at least as a dimer. The rather closer spacing of the A residues would, in that case, be determined by the geometry of the dimerization. Immunoprecipitations using V5-ZC3H11 co-expressed with ZC3H11-myc revealed no evidence for dimerization, although interference by the tags cannot be ruled out. It is possible that *in vivo*, interactions with other proteins serve to create ZC3H11 multimers.

### Conservation of ZC3H11 within the Kinetoplastidae

ZC3H11 is conserved throughout the Kinetoplastidae, as judged by reciprocal BLASTp analysis and alignments (Supplementary Figure S1). Nothing is known about the heat shock response in other salivarian trypanosomes, but some information is available for the more evolutionarily distant *Trypanosoma cruzi*. A proteome analysis found only minor changes after heat shock [47], and the level of *Tc*STI1 was also unchanged, both at the steady state level and for polysomal RNA [48]. *HSP70* mRNA is increased about two-fold after a 37°C heat shock of epimastigotes, and reporter experiments assigned responsibility to both 5′ and 3′-UTRs [49]. To see if the regulation by ZC3H11 might be conserved, we looked at the 3′-UTRs of the *T. cruzi* mRNAs encoding homologues of HSP70 (Tb11.01.3110), STI1 (Tb927.5.2940), DNAJ2 (Tb927.2.5160), HSP100 (Tb927.2.5980), FKBP (Tb927.10.16100), HSP110 (Tb927.10.12710) and DNAJ1 (Tb11.01.8750); all but the last one had extended (AUU) tracts. Thus at least as far as *T. cruzi*, the function of ZC3H11 might be conserved.

The *Leishmania ZC3H11* genes are not syntenic with those of trypanosomes, but nevertheless retain conserved sequence features (Supplementary Figure S1). Data for *L. infantum* indicate a 50% increase of *ZC3H11* mRNA during differentiation to axenic amastigotes, but no change was seen in intracellular amastigotes of *L. infantum* or *L. major* (http://tritrypdb.org/). It has long been known that *L. major* HSP70 and HSP83 - like the trypanosome counterparts - are preferentially synthesised after heat shock [50]. 3′-UTR analysis, however, suggests that the sequences required are not the same. Studies of *HSP70* in *L. infantum* showed that the 3′-UTR was responsible for mRNA stability after heat shock and that a region towards the 3′-end of it was required [24], [25]. There are however no (AUU) repeats in the entire 3′-UTR. *L. mexicana HSP70* (LmxM.28.2770) has UAUUUAUAUUAUAUU, but the function of this sequence is unknown. Similarly, the 3′-UTRs of the *Leishmania HSP83* mRNAs are well conserved between species, but bear no resemblance to those of trypanosome *HSP83*s. The *L. mexicana HSP83* mRNA lacks (AUU) repeats; a C/U-rich 150-nt region appears to be mainly responsible for regulation, perhaps via thermal melting [27]. The 3′-UTR of *L. braziliensis HSP100* (LbrM.29.1350), is devoid of (AUU) repeats, but *L. mexicana HSP100* (LmxM.08_29.1270) has the classical ARE, UAUUUAUUUAUU. The lack of conservation of the *L. mexicana* AU-rich sequences suggests that any function would be species-specific.

From this brief survey we suggest that ZC3H11 could be involved in regulating heat-shock genes in all trypanosomes. If it is involved in *Leishmania*, a different recognition sequence must be involved.

### Regulation by ZC3H11 after heat shock

After heat shock in procyclic forms, several mRNAs that bind to ZC3H11 are specifically stabilised. After an hour of heat shock, *HSP70* and *HSP83* mRNAs are also still actively translated (Figure 5D and [17], [18]). At the same time, heat-shock stress granules containing PABP and various translation initiation factors accumulate [17]. After 1 h heat shock, the amounts of phosphorylated ZC3H11 increased; only later, as cell viability decreased, did additional species appear which could either be dephosphorylated, or proteolytic fragments. We suggest that phosphorylation of ZC3H11 is increased after heat shock, resulting in increased ZC3H11 stability. The increase in ZC3H11 would stabilise existing *HSP* RNAs; since we saw no evidence for accumulation of ZC3H11 in granules, perhaps it prevents sequestration of target mRNAs.

The mammalian zinc-finger proteins TTP and BRF1 both induce degradation of bound mRNA containing AU-rich elements, and both are recruited to stress granules and P-bodies [51], [52]. Phosphorylation of TTP and BRF1 results in interaction with 14-3-3 isoforms, and consequent protein stabilisation, but at the same time, the functions of both factors in recruiting the mRNA degradation machinery, and their migration to stress granules, is impaired [51], [53], [54]. It is possible that phosphorylation of ZC3H11 acts in an analogous fashion.

### Roles of ZC3H11 in normal bloodstream and procyclic-form growth

ZC3H11 RNAi killed bloodstream forms, but not procyclics. It is quite possible that in procyclics, ZC3H11 is indeed essential, but the residual ZC3H11 after RNAi was sufficient for survival. Notably, we were unable to delete both *ZC3H11* genes although knockout constructs were able to integrate correctly into the genome (not shown). The most likely explanation for the difference in RNAi behaviour is that procyclics are routinely grown at a lower temperature (27°C), with a lower chaperone requirement: *ZC3H11* RNAi made procyclics hypersensitive to a temperature of 37°C.

The top 120 different mRNAs enriched in the ZC3H11 bound fraction represent, in total, only 300 RNA molecules per bloodstream-form trypanosome [14] so a role for the rather low amounts of ZC3H11 in regulating target RNA abundance or translation is quite possible. However, the overall role of ZC3H11 in bloodstream forms is unclear, especially as results from RNASeq and Northern blots were not always concordant. Many bound mRNAs were not affected by the RNAi, but this is not really surprising. All trypanosome 3′-UTRs are long enough to interact with multiple RNA-binding proteins, which are expected to influence the behaviour of the bound mRNAs in a combinatorial fashion. Many RNAs that either increased or decreased after *ZC3H11* RNAi were also not found bound to ZC3H11; perhaps the transcriptome after RNAi, at least in part, simply reflected the onset of growth inhibition. Further investigation - perhaps with transcriptomes taken at different times after *ZC3H11* RNAi induction, and also comparing various other growth-inhibitory conditions - will be required to ascertain which effects of ZC3H11 depletion are direct.

### The chaperone cycle and ZC3H11 specificity

Chaperones assist in the folding of the nascent polypeptide chains, refold denatured proteins, and regulate the function of bound proteins. The *T. brucei* genome encodes large numbers of predicted or known chaperone-pathway proteins [55]: our domain searches revealed 5 Hsp70s, 11 Hsp20s, 35 petidyl-prolyl *cis-trans* isomerases, and 73 DnaJ-domain proteins. Among these, ZC3H11 mRNA binding was highly biased towards gene products that are required, not just for constitutive protein folding, but for recovery from stress. Notably, the complete refolding cycle [56], [57] was represented: the major cytosolic HSP70; three DnaJ-domain proteins (HSP40s) and three Hsp20s, two TPR-repeat-containing peptidyl-prolyl *cis-trans* isomerases, the major cytosolic Hsp90 homologue HSP83, HSP100, HSP110 and the regulator STI1. ZC3H11 bound mRNA encoding mitochondrial HSP60, but not to any of those encoding the cytosolic TriC complex. ZC3H11 also bound to several mRNAs encoding proteins of unknown function, and without recognisable domains, some of which were elevated after heat shock, but are not required for normal growth [58]. We speculate that some of these could be involved in the heat-shock response.

### Does the trypanosome heat-shock response confer a selective advantage?

Since many of the chaperones that are involved in the trypanosome heat-shock response are essential at normal growth temperatures, it has hitherto not been possible to tell whether the heat-shock response itself (as opposed to the constituent proteins) has any role in Kinetoplastid survival. Indeed, in *Leishmania*, the steady-state levels of two of the major proteins, HSP83 and HSP70, are so high that they are unaffected by the transient increases in their synthesis that occurs after heat shock [59]. We have now found that in procyclic forms, depletion of ZC3H11 not only prevented the continued synthesis of HSP70 and HSP83, and the stabilisation of several other chaperone mRNAs, after a 41°C heat shock, but also severely impaired the ability of the trypanosomes to recover when returned to 27°C. This shows, for the first time, that a Kinetoplastid heat-shock response *per se* protects the parasite from short periods at an elevated temperature.

At first sight, the temperatures that are required for the trypanosome heat shock response seem un-physiologically high. For bloodstream forms, an increase *HSP70* mRNA was seen only at 41.5°C and above [60]. Such temperatures are rare in humans and many of the wild ungulates that are natural hosts for trypanosomes. Running African gazelles are, however, able to survive body temperatures of 43°C without ill-effects [6], and trypanosomiasis in gazelles can cause fever temperatures of 43°C [7]. Procyclic trypanosomes show a heat-shock response at and above 37°C. Their host Tsetse flies are not found in environments in which the maximum air temperature exceeds 41°C [8]; Tsetse rest during the hottest part of the day [8] and feed preferentially on the lower (more shady) parts of animals [61]. Nevertheless, 10% of a population of *Glossina pallipides* were able to survive an hour at 41°C, and during gradual heating, some can survive up to 44°C; [62]. Overall, therefore, it seems that trypanosome heat-shock responses have evolved such that they are induced at 1–2°C below the maximum temperatures that are likely to be experienced in the relevant host. The heat-shock response is therefore tuned to enhance parasite survival under field conditions.

## Materials and Methods

### Cells and plasmids

Details of all plasmids and oligonucleotides are provided in Supplementary Table S2. All experiments were done with Lister 427 monomorphic procyclic or bloodstream form parasites expressing the Tet-repressor [63]. Procyclic forms were grown in MEM-Pros medium at 27°C (unless stated otherwise) at densities lower than 8×10^6^ cells/ml. The bloodstream stage parasites were cultivated in HMI-9 medium in an incubator at 37°C with 5%CO_2_ at densities lower than 1.5×10^6^ cells/ml.

Additionally, various stable cell lines were created with constitutive (CAT reporter/V5) or tetracycline-inducible expression (RNAi, ectopic expression).

For the tethering assays, cell lines constitutively expressing CAT reporter with actin 3′-UTR or boxB actin 3′-UTR were co-transfected with an inducible lambdaN-ZC3H11-myc fusion protein.

Fragments of the ZC3H11 open reading frame (first 104a.a., 119a.a., 136a.a., 199a.a., with or without a C→S mutation in the zinc finger) were cloned into pQEA38 and expressed as His10-fusions in *E.coli* (strain Rosetta, D3 pLysS, Novagen). The full-length ZC3H11 open reading frame was cloned in pET-trx1b (pHD2222) and co-expressed with the groES-groEL-tig chaperone system (Takara's Chaperone Plasmid, pG-Tf2) in *E.coli* (strain Tuner, Novagen). Buffer in protein samples was exchanged to binding buffer (20 mM Tris-HCl, pH 8.0, 50 mM NaCl, 100 µM ZnCl_2_) using Amicon Ultracentrifugal filter 10 kDA NMWCO columns. Protein aliquots were supplemented with 10% glycerol (final concentration) and stored at −80°C.

### Protein detection and manipulation

Dephosphorylation assays [64] and co-immunoprecipitation assays [65] were done as previously described. Phosphatase inhibitors used were Sodium Orthovanadate (2 mM) and Sodium Fluoride (8 mM). For co-immunoprecipitation with V5-ZC3H11, 4×10^7^ procyclic trypanosomes were pre-treated for 1 h with the proteasome inhibitor MG-132 (Calbiochem) at a concentration of 10 µg/ml.

Cells were lysed in hypotonic buffer (10 mM NaCl, 10 mMTris-Cl pH 7.5, 0.1% NP40 with complete protease inhibitor (Roche), and precipitation was with anti-myc (Biomol) -coupled beads after the salt was adjusted to 150 mM NaCl.

Proteins were detected by Western blotting. Antibodies used were to the V5 tag (AbD seroTec, 1∶1000), the Myc tag (Santa Cruz Laboratories, 1∶1000), aldolase (rabbit, 1∶50000 [66]) Detection was done using ECL solutions (GE Healthcare). Chloramphenicol acetyltransferase was measured in a kinetic assay involving partition of ^14^C-buturyl chloramphenicol from the aqueous to the organic phase of scintillation fluid [67].

### Effect of heat shock on protein synthesis

Trypanosomes were subjected to heat-shock at 41°C (for 1 hour) in a water bath and harvested immediately for RNA and Western blot. To measure protein synthesis, 2×10^6^ cells were pelleted, resuspended in 500 µl of MEM lacking methionine. After 15 mins, [^35^S] methionine (Amersham, 20 µCi) was added and the cells were incubated at 27°C for 20 min. Pelleted cells were washed once (1× PBS+0.5%Glucose) then resuspended in Laemmli sample buffer and subjected to SDS-PAGE. The gel was fixed in 10% acetic acid, 30% methanol solution in water for 45 min, stained with Coomassie followed by de-staining in water. The gel was then incubated for 45 min in En3Hance (Amersham), washed in water for another 45 mins, dried and exposed for autoradiography.

### RNA co-precipitation

2*10^8^ procyclic cells were UV-cross-linked (400 mJ/cm^2^) prior to freezing in liquid nitrogen. Frozen pellets were resuspended in 500 µl hypotonic buffer (10 mM Tris pH 7.5; 10 mM NaCl; 0.1% IGEPAL) containing protease inhibitor (complete mini EDTA free; Roche) and 8 mM VRCs (Sigma) and 800 u RNasin (Promega). Lysis was completed by passing 15 times through a 27G needle. After pelleting insoluble debris (at 6000 g) and adjusting to 150 mM NaCl, the protein was allowed to bind for one hour at 4°C to anti-myc coupled agarose (Biomol). The agarose was then washed 4 times at 4°C with IPP150 (10 mM Tris pH 7.5; 150 mM NaCl; 0.1% IGEPAL). Cross-linked protein was digested with 20 µg proteinase K at 42°C for 15 min, and RNA was isolated from both pellet and unbound fractions using Trifast (Peqlab).

### RNA and transcriptome analysis

Total RNA was extracted using Trifast (Peqlab). Blotted RNA was detected by hybridization with radioactive probes (see Supplemental Table S2). For microarray analysis of cells with *ZC3H11* RNAi, we used oligonucleotide arrays (mostly one oligonucleotide per open reading frame) from NIAID and TIGR. They were hybridised with cDNA made from total RNA from either bloodstream forms expressing the repressor, or cells with RNAi after one day of tetracycline induction, as previously described [68].

For analysis of RNA bound to ZC3H11, the unbound RNA fraction was treated with the Ribominus kit (Invitrogen) to remove ribosomal RNAs. Both eluate and unbound RNAs were then subjected to deep sequencing, and reads aligned to the genome as previously described [14]. To normalise the data we calculated the total number of reads in a set of unique genes [69], with the major HSP70, GPEET procyclin and EP procyclin added. This number was used in order to calculate the reads per million total reads.

To extract all known 3′-UTRs, we used all of our available sequence sets and extracted the reads that failed to align to the genome. From these, we selected those that contained at least five contiguous T residues at the start of the read. After removal of the T's, we re- aligned the reads to the genome. For each ORF, the most abundantly aligned sequence was assigned as the major polyadenylation site; if two gave equal read densities, the proximal one was used. The output is available on request. For mRNAs that were classified as strongly (3×) enriched in the ZC3H11-bound sample, whenever 3′-UTRs were missing or too short we extracted them manually using published information and processing sites annotated in the TritrypDB database, or simply used 200 bp downstream of an ORF.

MDscan [70] was used to search for motifs in the 3′-UTRs of mRNAs that were at least 3-fold enriched in the eluate, using the top 10 bound mRNA's as seeds. A similar motif search was also done using MotifSampler [71] with a 3rd order background model constructed from all known 3′-UTRs. The motif with the highest representation in the MotifSampler output, and highest log likelihood score, was identified as a potential binding motif of ZC3H11, and it was in agreement with the MDscan motif. Next, FIMO [72] was used to scan all known 3′-UTRs using the model of the motif we found, and only hits with a q-value<0.01 were retained.

RNASeq analysis was performed on poly(A)+ mRNA, except for the procyclic wild-type sample, which was part of another study and was rRNA depleted, not poly(A) selected. Previous results with bloodstream forms indicated that at steady state, these populations are similar (R = 0.96) [14]. The RNA was fragmented before cDNA synthesis, using the standard Illumina protocol. Alignment and normalisation were as described above. The relevant Supplementary Table S1 sheets (3–5) include all unique genes with rpm of at least 10 in each of the relevant datasets. Since these experiments were performed only once, we focus in our discussions on results that were also verified by other means.

### In vitro RNA-protein binding assay

Oligoribonucleotides (50 pmol, Biomers, 50 µl reaction) were 5′-end labelled with 50 µCi of gamma-[^32^P] ATP and 20 units of T4 polynucleotide kinase (New England Biolabs) for 37°C for 30 min, then purified using the QIAquick Nucleotide Removal Kit (QIAGEN). Recombinant proteins (100 pmol) were incubated for 30 min at room temperature in binding buffer (20 mM Tris-HCl, pH 8.0, 50 mM NaCl, 10 µM ZnCl_2_, 0,01% IGEPAL CA-630, 0.1 mg/ml of tRNA, 10 µg/ml of heparin) with 1 pmol radiolabeled RNA, with or without 5- and 20-fold excess competitor RNA [73]. Loading dye was added and the samples were run on 5% non-denaturing polyacrylamide gels, (0.3× tris-borate buffer +100 µM ZnCl_2_; 30 min at 300 V) which were then dried and analysed using a phosphorimager. For quantitative analyses, varying concentrations of protein were incubated with 10 pmol labelled RNA [73].

## Supporting Information

Figure S1 A. Alignment of amino-acid sequences of selected Kinetoplastid ZC3H11s. The alignment was done using ClustalW (Slow, accurate, Gonnet), in the DNAStar package. Identical amino acids are shadowed in black and conserved amino acids in grey. Groups used were : (DE), (HKR), (AGILV), (NQ), (FWY), (ST), (P), (CM). The CCCH motif is in pink. The codes for conserved residues are listed at the bottom. Arrows indicate clone boundaries: a) N-terminus of the C-terminal fragment used in the tethering assay; b) C terminus of ZC3H11-104 used for RNA-binding assays; c) C-terminus of ZC3H11-119 used for RNA-binding assays; d) C-terminus of the N-terminal fragment used in the tethering assay. B. Location of ZC3H11-myc in procyclic forms. Results are shown for normal cells (not expressing myc-tagged protein) and cells expressing ZC3H11-myc grown at the normal temperature (27°C) or subjected to a one-hour heat shock at 41°C. DIC - differential interference contrast image.
(PDF)

Figure S2 Binding of the ZC3H11 zinc finger to AUU repeats. **A.** Recombinant trx-ZC3H11 (trx-Z) and His-tagged N-terminal fragments (indicated above by their sizes) were purified, separated by denaturing SDS-PAGE and stained with Coomassie blue. Arrows indicate the recombinant proteins. M: markers. **B.** Recombinant trx-ZC3H11 and His-tagged N-terminal fragments were separated by native PAGE and stained with Coomassie blue. Bovine serum albumin (BSA) served as a control. **C.** The His-tagged N-terminal 119mer of ZC3H11 (100 pmol total protein, Z-104) was incubated with 1 pmol radioactively labelled probe, with 5 µg/ml heparin, then separated by native PAGE. The phosphorimager output is shown. Radioactive RNA probes were: UAU: U(UAU)7U; UAUU: (UAUU)5UAU; UCU: U(UCU)7U; U: (U)23; C: (C)23; A: (A)23. Arrows indicate unbound probe (u), a UAU or UAUU-specific complex (s2) and a different UCU or poly(U)-specific complex (n). **D.** The His-tagged N-terminal ZC3H11 119mer (100 pmol total protein) was incubated with 1 pmol radioactively labelled U(UAU)7U in the presence of 5 µg/ml heparin and competing oligonucleotides in 5 or 20-fold excess. U(UAU)7U in the presence of 5 µg/ml heparin and competing oligonucleotides in 5 or 20-fold excess. **E.** Competition assay for both labelled probes in the presence of 10 mg/ml heparin. The non-specific band persisted. **F.** Titration of increasing amounts of recombinant trx-ZC3H11 against limiting amounts of U(UAU)_7_U RNA probe. The protein was incubated with radioactively-labelled probe then separated by native PAGE. The phosphorimager output is shown. Approximate maximum and minimum protein levels (in Molar units) are shown at the top. A quantitation of radioactivity in the well relative to unbound probe (u) shown on the right. 50% binding of 10 pM probe was seen with about 10 nM recombinant ZC3H11-119. Since, however, we do not know how much of the recombinant protein is properly folded, and several proteins may bind to a single probe, this value cannot be taken to be a dissociation constant.
(PDF)

Figure S3 Effects of ZC3H11 depletion and heat shock on the transcriptome. The graphs show reads per million reads for individual unique open reading frames (ORFs). All data were normalised to one set of unique genes, according to Siegel et al. (Nucleic Acids Res. 38, 4946–57) and the most recent genome annotations. For each plot, genes with less than 10 reads per million in either of the conditions plotted were removed. **A.** Poly(A)+ mRNA was prepared from bloodstream-form trypanosomes with *ZC3H11* RNAi, induced for 24 h. The results were plotted against those for wild-type bloodstream forms, as previously published (Manful et al., RNA 17, 2039–2047) but re-normalised for the relevant gene set. The number of genes analysed is in the top left-hand corner, and a linear regression formula at the bottom right. The pink diagonal represents perfect correlation. **B.** As in (A), but only genes with less than 1000 reads per million are plotted. The linear regression line is shown in black. **C.** Northern blots for bloodstream forms with and without *ZC3H11* RNAi. Results from 3 different blots are shown. **D.** Poly(A)+ mRNA was taken from procyclic trypanosomes heated to 41°C for 1 h, and compared to a dataset for rRNA-depleted RNA from procyclic forms grown at 27°C. Previous results have shown that at steady state, there is little difference between rRNA-depleted and poly(A)+ transcriptomes (Manful et al., RNA 17, 2039–2047). Other details are as in (A). **E.** Poly(A)+ mRNA was taken from procyclic trypanosomes heated to 41°C for 1 h; wild-type trypanosomes compared with parasites with *ZC3H11* RNAi. Other details as in (A).
(PDF)

Figure S4 *CAT* reporter mRNAs. **A.** Constructs used to express *CAT* with various 3′-UTRs. All constructs are designed for integration in the beta tubulin locus. The CAT open reading frame (grey box) is preceded by the EP 5′ region and followed by a linker (in the control cell line) or the 3′-UTR or 3′-UTR fragment of interest (marked in light grey). This is followed by a truncated actin intergenic region still containing a possible poly(A) addition site (pA), the polypyrimidine tract (pY) and splice acceptor dinucleotide (AG). The expected reporter mRNA sizes (including the spliced leader sequence, without considering a poly(A) tail) are given below each construct. **B.** Northern blot with total RNA of CAT reporter cell lines and induction of ZC3H11 RNAi, hybridised with a *CAT*, *ZC3H11* and *HSP70* probes with *7SL* used as a control. **C.** RNA from four different reporter cell lines (as indicated) was incubated with oligo d(T) and RNase H to remove poly(A) tails (Schwede et al, Nucleic Acids Res. 37, 5511–5528), before Northern blotting and hybridising with a *CAT* probe as in (B). The bands are of the expected sizes, **D.** AU-rich elements can effect reporter expression in a non-ZC3H11 dependent manner. Details as in Figure 4D.
(PDF)

Figure S5 Effects of heat shock (1 h, 41°C) on procyclic trypanosome mRNAs with or without *ZC3H11* RNAi. Various blots from different experiments are shown, with quantitation relative to rRNA (marked with *) or the *7SL* RNA. **A.** *ZC3H11*, *HSP70* and *HSP83* mRNA levels from the same experiment were quantified relative to the rRNA (methylene blue staining). **B.** *HSP100* (Tb927.2.5980) and *DNAJ2* (Tb927.2.5160) **C.** *HSP110* (Tb927.10.12710) **D.** *FKBP* (Tb927.10.16100) **E.** Alpha tubulin, *TUB* **F.** Glycerol-3 phosphate dehydrogenase (GPDH, Tb927.8.3530). Due to a transfer problem at the *7SL* signal, ribosomal RNA was used for normalization.
(PDF)

Table S1 RNASeq datasets and analyses. Sheet 1: RNASeq data for mRNAs bound to ZC3H11. In addition to the values for ZC3H11 binding (RNA bound/FT rpkm) the results from the other sheets are provided. Sheet 2: Motifs enriched in the 3′-UTRs of mRNAs bound to ZC3H11. Sheet 3: RNASeq results for poly(A)+ RNA of bloodstream-form trypanosomes after 1 day of induction ZC3H11 RNAi, compared with wild-type. Sheet 4: RNASeq results for poly(A)+ RNA of procyclic-form trypanosomes after one hour at 41°C, compared to cells kept at 27°C. Sheet 5: RNASeq results for poly(A)+ RNA of procyclic-form trypanosomes depleted of ZC3H11, after one hour at 41°C, compared to similarly heat-shocked cells without RNAi. Sheet 6: RNASeq results for the part of the ZC3H11 gene that is not included in the dsRNA used for RNAi. This gene was removed from Sheets 3 and 5. Sheet 7: Gene functional classes enriched under particular conditions: p-values are shown (see methods section). For each sheet, further details are provided in text boxes.
(XLSX)

Table S2 Plasmids and oligonucleotides used in this paper.
(DOCX)
