# Supplementary material for: Post-Transcriptional Regulation of the Trypanosome Heat Shock Response by a Zinc Finger Protein
Source: PLoS Pathog. 2013 Apr 4;9(4):e1003286. doi: 10.1371/journal.ppat.1003286 (PMC3616968; doi:10.1371/journal.ppat.1003286)
Supplement: Figure S1 — A. Alignment of amino-acid sequences of selected Kinetoplastid ZC3H11s. The alignment was done using ClustalW (Slow, accurate, Gonnet), in the DNAStar package. Identical amino acids are shadowed in black and conserved amino acids in grey. Groups used were : (DE), (HKR), (AGILV), (NQ), (FWY), (ST), (P), (CM). The CCCH motif is in pink. The codes for conserved residues are listed at the bottom. Arrows indicate clone boundaries: a) N-terminus of the C-terminal fragment used in the tethering assay; b) C terminus of ZC3H11-104 used for RNA-binding assays; c) C-terminus of ZC3H11-119 used for RNA-binding assays; d) C-terminus of the N-terminal fragment used in the tethering assay. B. Location of ZC3H11-myc in procyclic forms. Results are shown for normal cells (not expressing myc-tagged protein) and cells expressing ZC3H11-myc grown at the normal temperature (27°C) or subjected to a one-hour heat shock at 41°C. DIC - differential interference contrast image. (PDF) [file ppat.1003286.s001.pdf]

A

```
Tb927.5.810          MSTATSAPVVESMSDTVVCPQLSRHSTSLEELYTIGYNRSHSPSCFSSAGCEEEEGQKN[illegible]LAERYKTKLCKNFVQYGTCP 80
TcIL3000.0.37550     MPAALSKPAKMMIMSDGQCEVQSSRSCSLSQSIVPCGSYTGSP---SVAEMDEEGHAKV[illegible]LAERYKTKYCNNFVINGACP 77
TvY486_0500320       -MAFDSSSVTSLQSLSAPDVLPPSLSVDKPHFFADYNADFN-------------KHGKT[illegible]LAERYKTKLCKNFMELSFCP 66
Tc00.1047053504929.5 --------MTSMHCTLVPTPPPPFYGVCMEDTMSLGYACAS--------GVESDMAAKP[illegible]LAERYKTKFCRN[illegible]VLTGICP 64
LmxM.34.1040         --------------------MSKNTGSVTEQHKRISHAAGD-----------NNEEEKH[illegible]LAGRYKTKLCKN[illegible]VAKGECP 49

                                         a    b                c         d
Tb927.5.810          YDIRCMFAHGEEELRTAEMNIMDGLVTK[illegible]AITSFQ[illegible]LWHRAVSASSGSKHHSSH-----VANRRGVMTTATTTTTAG--- 152
TcIL3000.0.37550     YDTRCMFAHSAEELRTAEMNIADGLITMEAIRRFQRLLHQVTRSSRFARLRPVR-----TSAPWAMVPAASIGPAAMP-- 150
TvY486_0500320       YGFICMFAHGEEELRTPKMN[illegible]EDGLSTEESIRVFQRMWYKVAFQQQHSAVQN----------SVGFLWPFTCTSLMP--- 133
Tc00.1047053504929.5 YQRRCMFAHGDHELRTPEMNIRDGLVTEEAIRLFRRQFLSDICAFDRLRRR-------------GFPAPPYFYHAMG--- 128
LmxM.34.1040         YDVRCMFAHGEDELRTSDDNIRDGLVTEEAIKDFQRQQNQAKRRAAFAAAREHNNHDHSHSHNHSHTHNHTVRPVSHRNA 129

Tb927.5.810          -------GSTDAAMHICRRMQRVVAAAAAAVPCGGRRVVS---------GSIRIRGCVRHNPYCHNI[illegible]PTVSRYYSTMPD 216
TcIL3000.0.37550     ------HAYHDLRQSSAKGICERAGCVAA--------------------ATPMAGVRYTHNPYRCDI[illegible]PLLSRLYSKLPD 204
TvY486_0500320       ---------PPPYHHHVPYFAQIPVKSTT--------------------SFPPPAARYRHDPYCAE[illegible]IPPESRFYSRLPD 184
Tc00.1047053504929.5 -----------VCMSDCG-----VP------------------------VRMAPAKIYTHNPYKSDIIPLQNRLYSQPPA 168
LmxM.34.1040         YEIEENVESNEVYHNRHRHHNVRLHPQQESVSIPQPQQQQQQQHEEQFQLQSAVVQYYTHNPYVFD[illegible]IPAVARL[illegible]PVYHD 209

Tb927.5.810          VFTRPYSKSDSCGNEAITEGNAWTYANEEGNEKETPAMISSGVPELTAWKRFNYVAERSQCTSVPEQNLPCADFAAIKES 296
TcIL3000.0.37550     MFVVPCNRVPDSGSELAQLPEERLSERILDGDE--------TVPAFTERANAQQEQLRCDSDARCHEALFLPESLPSDEP 276
TvY486_0500320       AALDSQEAR--------------------------------NKHCYCSRAQSDNGKEGALCTASPASECFSQFQLTTLQL 232
Tc00.1047053504929.5 LAMMTAAT---------------------------------VMPQYYEEG----TMCRSSCDPSEHQDDFEPCVEIVSSP 211
LmxM.34.1040         VV----------------------------------EEGCWYEEEPAPAQEDYYFPSSDMMMPLVPSLVTQQYLPYPHQQ 255

Tb927.5.810          NNTGGNASGVDVENV--DDAEHSTCSS[illegible]GNESGYFCRASRSASCGEQSQSHLKREGNEGRGEGLHMFLSL 364
TcIL3000.0.37550     DVACTPDSGSEEVDG--[illegible]VGDRSVCSS[illegible]GNESGDVHNSPTYASHFVKG--SLRTVGRGVSEESSHTDLYL 342
TvY486_0500320       QNSENEPQLLPHIRS--DDGDSSLCSS[illegible]GSHSGLSRSMSVTNSLAKRS----ESLSTYHEVEVIQ 291
Tc00.1047053504929.5 RCYACNES---------DDAEPSACSS[illegible]GSDSEDNRQSTPCKEYINES---MPSEEVMSGEAALQ 264
LmxM.34.1040         CGKQFETVASASTYSGE[illegible]AMYGHSESSASSVYRSVARCTAAELPSNSDIPAMVMEP 311
```

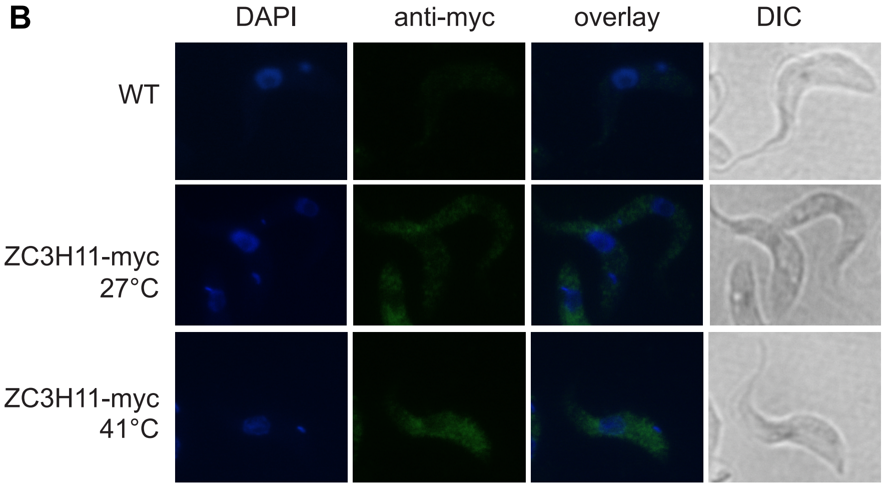

Supplementary Figure S1

A. Alignment of amino-acid sequences of selected Kinetoplastid ZC3H11s. The alignment was done using ClustalW (Slow, accurate, Gonnet), in the DNAStar package. Identical amino acids are shadowed in black and conserved amino acids in grey. Groups used were : (DE), (HKR), (AGILV), (NQ), (FWY), (ST), (P), (CM). The CCCH motif is in pink. The codes for conserved residues are listed at the bottom. Arrows indicate clone boundaries: a) N-terminus of the C-terminal fragment used in the tethering assay; b) C terminus of ZC3H11-104 used for RNA-binding assays; c) C terminus of ZC3H11-119 used for RNA-binding assays; d) C-terminus of the N-terminal fragment used in the tethering assay.

B. Location of ZC3H11-myc in procyclic forms. Results are shown for normal cells (not expressing myc-tagged protein) and cells expressing ZC3H11-myc grown at the normal temperature (27°C) or subjected to a one-hour heat shock at 41°C. DIC - differential interference contrast image.
