# Supplementary material for: Post-Transcriptional Regulation of the Trypanosome Heat Shock Response by a Zinc Finger Protein
Source: PLoS Pathog. 2013 Apr 4;9(4):e1003286. doi: 10.1371/journal.ppat.1003286 (PMC3616968; doi:10.1371/journal.ppat.1003286)
Supplement: Figure S2 — Binding of the ZC3H11 zinc finger to AUU repeats. A. Recombinant trx-ZC3H11 (trx-Z) and His-tagged N-terminal fragments (indicated above by their sizes) were purified, separated by denaturing SDS-PAGE and stained with Coomassie blue. Arrows indicate the recombinant proteins. M: markers. B. Recombinant trx-ZC3H11 and His-tagged N-terminal fragments were separated by native PAGE and stained with Coomassie blue. Bovine serum albumin (BSA) served as a control. C. The His-tagged N-terminal 119mer of ZC3H11 (100 pmol total protein, Z-104) was incubated with 1 pmol radioactively labelled probe, with 5 µg/ml heparin, then separated by native PAGE. The phosphorimager output is shown. Radioactive RNA probes were: UAU: U(UAU)7U; UAUU: (UAUU)5UAU; UCU: U(UCU)7U; U: (U)23; C: (C)23; A: (A)23. Arrows indicate unbound probe (u), a UAU or UAUU-specific complex (s2) and a different UCU or poly(U)-specific complex (n). D. The His-tagged N-terminal ZC3H11 119mer (100 pmol total protein) was incubated with 1 pmol radioactively labelled U(UAU)7U in the presence of 5 µg/ml heparin and competing oligonucleotides in 5 or 20-fold excess. U(UAU)7U in the presence of 5 µg/ml heparin and competing oligonucleotides in 5 or 20-fold excess. E. Competition assay for both labelled probes in the presence of 10 mg/ml heparin. The non-specific band persisted. F. Titration of increasing amounts of recombinant trx-ZC3H11 against limiting amounts of U(UAU)7U RNA probe. The protein was incubated with radioactively-labelled probe then separated by native PAGE. The phosphorimager output is shown. Approximate maximum and minimum protein levels (in Molar units) are shown at the top. A quantitation of radioactivity in the well relative to unbound probe (u) shown on the right. 50% binding of 10 pM probe was seen with about 10 nM recombinant ZC3H11-119. Since, however, we do not know how much of the recombinant protein is properly folded, and several proteins may bind to a single probe, this value cannot be take [file ppat.1003286.s002.pdf]

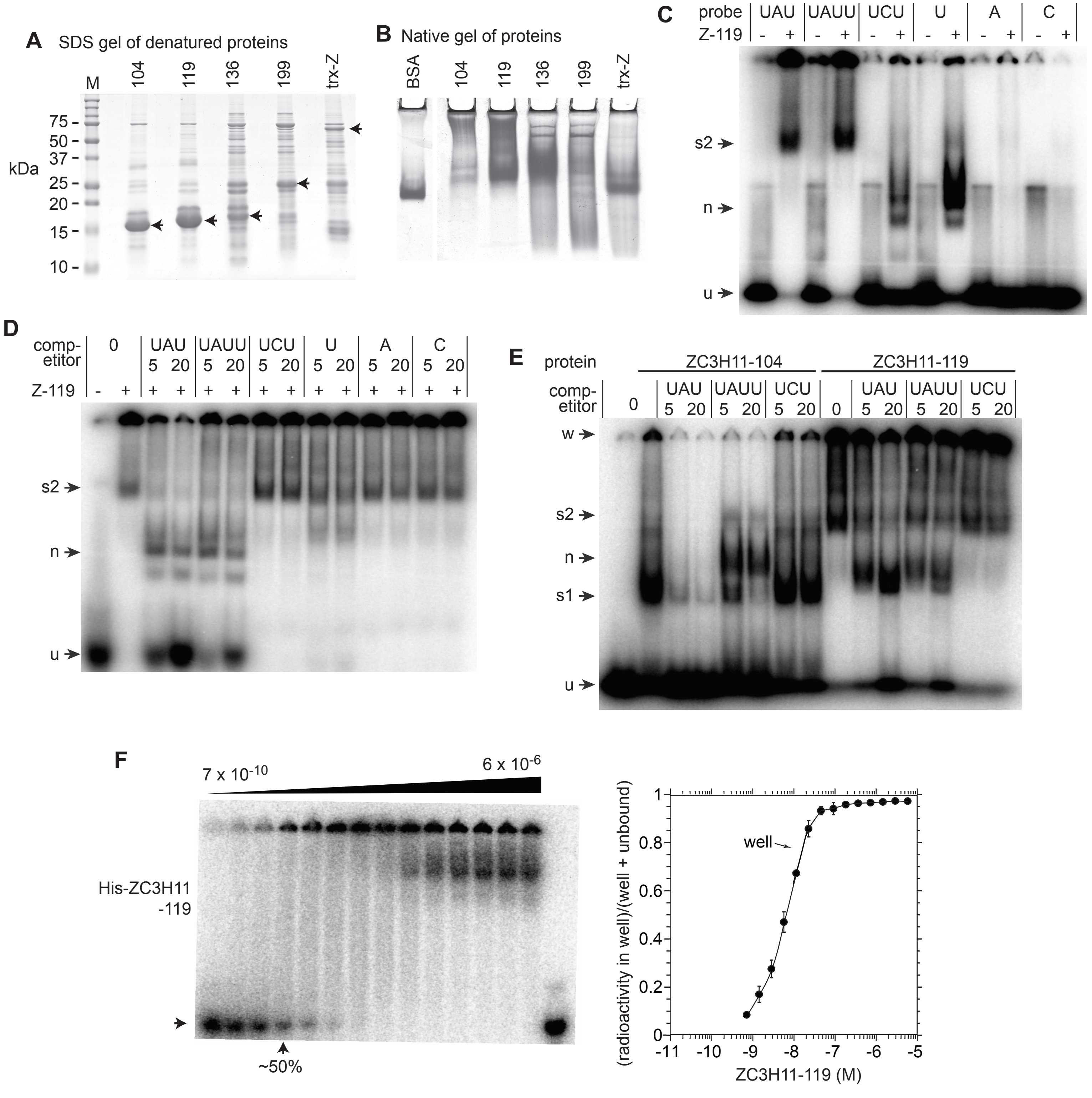

Supplementary Figure S2

Binding of the ZC3H11 zinc finger to AUU repeats

A. Recombinant trx-ZC3H11 (trx-Z) and His-tagged N-terminal fragments (indicated above by their sizes) were separated by denaturing SDS-PAGE and stained with Commassie blue. Arrows indicate the recombinant proteins. M: markers.

B. Recombinant trx-ZC3H11 and His-tagged N-terminal fragments were separated by native PAGE and stained with Commassie blue. Bovine serum albumin (BSA) served as a control.

C. The His-tagged N-terminal 119mer of ZC3H11 (100 pmol total protein, Z-104) was incubated with 1 pmol radioactively labelled probe, with 5µg/ml heparin, then separated by native PAGE. The phosphorimager output is shown. Radioactive RNA probes were: UAU: U(UAU)7U; UAUU: (UAUU)5UAU; UCU: U(UCU)7U; U: (U)23; C: (C)23; A: (A)23. Arrows indicate unbound probe (u), a UAU or UAUU-specific complex (s2) and a different UCU or poly(U)-specific complex (n).

D. The His-tagged N-terminal ZC3H11 119mer (100 pmol total protein) was incubated with 1 pmol radioactively labelled U(UAU)7U in the presence of 5µg/ml heparin and competing oligonucleotides in 5 or 20-fold excess.

E. Competition assay for both labelled probes in the presence of 10mg/ml heparin. The non-specific band persisted.

F. Titration of increasing amounts of recombinant trx-ZC3H11 against limiting amounts of U(UAU)7U RNA probe. The protein was incubated with radioactively-labelled probe then separated by native PAGE. The phosphorimager output is shown. Approximate maximum and minimum protein levels (in Molar units) are shown at the top. A quantitation of radioactivity in the well relative to unbound probe (u) shown on the right. 50% binding of 10 pMol probe was seen with a concentration of about 10 nMol recombinant AC3H11-119. Since, however, we do not know how much of the recombinant protein is properly folded, and several proteins may bind to a single probe, this value cannot be taken to be a dissociation constant.
