# Supplementary material for: Post-Transcriptional Regulation of the Trypanosome Heat Shock Response by a Zinc Finger Protein
Source: PLoS Pathog. 2013 Apr 4;9(4):e1003286. doi: 10.1371/journal.ppat.1003286 (PMC3616968; doi:10.1371/journal.ppat.1003286)
Supplement: Figure S3 — Effects of ZC3H11 depletion and heat shock on the transcriptome. The graphs show reads per million reads for individual unique open reading frames (ORFs). All data were normalised to one set of unique genes, according to Siegel et al. (Nucleic Acids Res. 38, 4946–57) and the most recent genome annotations. For each plot, genes with less than 10 reads per million in either of the conditions plotted were removed. A. Poly(A)+ mRNA was prepared from bloodstream-form trypanosomes with ZC3H11 RNAi, induced for 24 h. The results were plotted against those for wild-type bloodstream forms, as previously published (Manful et al., RNA 17, 2039–2047) but re-normalised for the relevant gene set. The number of genes analysed is in the top left-hand corner, and a linear regression formula at the bottom right. The pink diagonal represents perfect correlation. B. As in (A), but only genes with less than 1000 reads per million are plotted. The linear regression line is shown in black. C. Northern blots for bloodstream forms with and without ZC3H11 RNAi. Results from 3 different blots are shown. D. Poly(A)+ mRNA was taken from procyclic trypanosomes heated to 41°C for 1 h, and compared to a dataset for rRNA-depleted RNA from procyclic forms grown at 27°C. Previous results have shown that at steady state, there is little difference between rRNA-depleted and poly(A)+ transcriptomes (Manful et al., RNA 17, 2039–2047). Other details are as in (A). E. Poly(A)+ mRNA was taken from procyclic trypanosomes heated to 41°C for 1 h; wild-type trypanosomes compared with parasites with ZC3H11 RNAi. Other details as in (A). (PDF) [file ppat.1003286.s003.pdf]

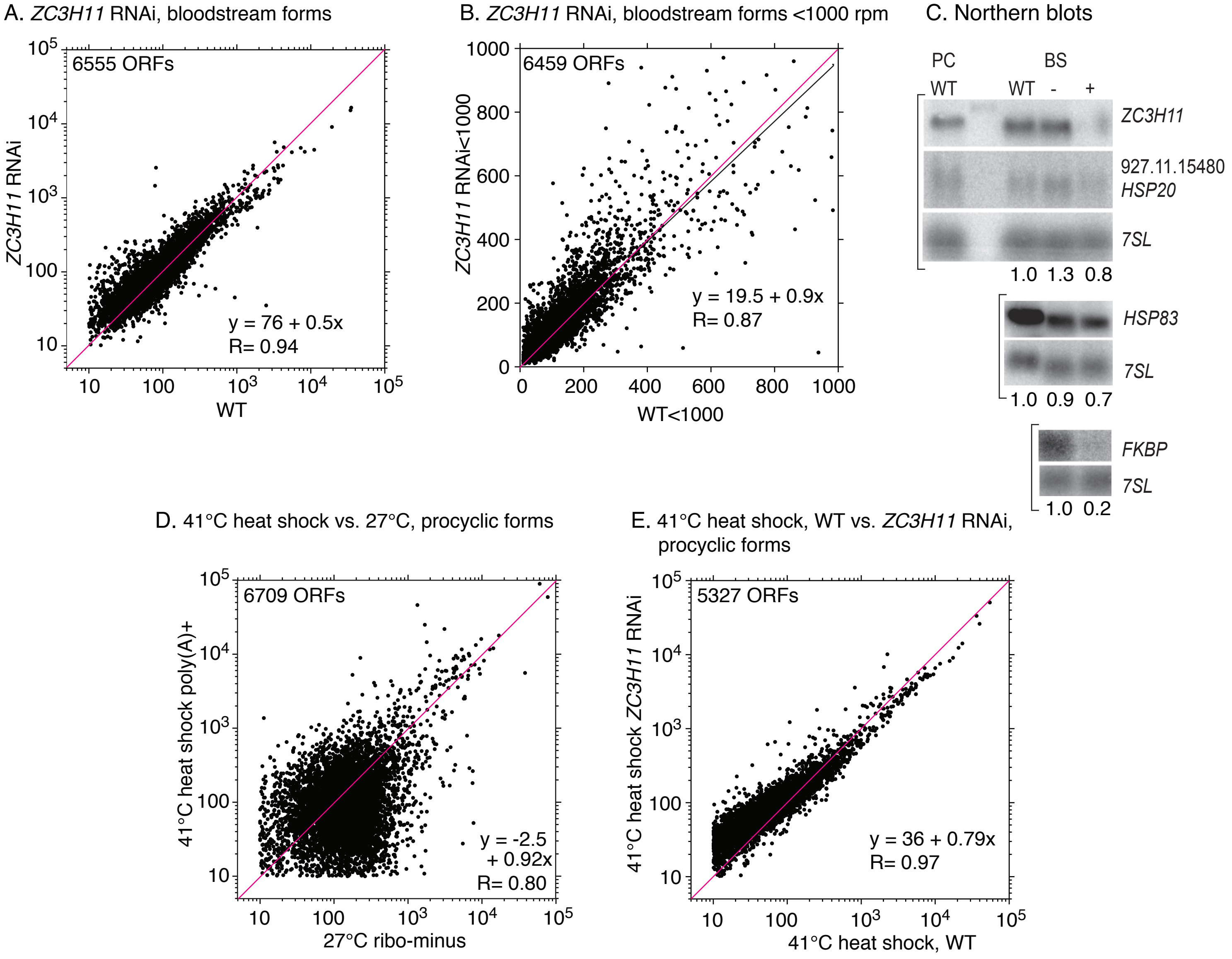

Supplementary Figure S3

Effects of ZC3H11 depletion and heat shock on the transcriptome.

The graphs show reads per million reads for individual unique open reading frames (ORFs). All data were normalised to one set of unique genes, according to Siegel et al. (Nucleic Acids Res. 38, 4946-57) and the most recent genome annotations. For each plot, genes with less than 10 reads per million in either of the conditions plotted were removed.

A. Poly(A)+ mRNA was prepared from bloodstream-form trypanosomes with *ZC3H11* RNAi, induced for 24h. The results were plotted against those for wild-type bloodstream forms, as previously published (Manful et al., RNA 17, 2039-2047) but re-normalised for the relevant gene set. The number of genes analysed is in the top left-hand corner, and a linear regression formula at the bottom right. The pink diagonal represents perfect correlation.

B. As in (A), but only genes with less than 1000 reads per million are plotted. The linear regression line is shown in black.

C. Northern blots for bloodstream forms with and without *ZC3H11* RNAi. Results from 3 different blots are shown, with quantitation of the chaperone mRNAs beneath.

D. Poly(A)+ mRNA was taken from procyclic trypanosomes heated to 41°C for 1h, and compared to a dataset for rRNA-depleted RNA from procyclic forms grown at 27°C. Previous results have shown that at steady state, there is little difference between rRNA-depleted and poly(A)+ transcriptomes (Manful et al., RNA 17, 2039-2047). Other details are as in (A).

E. Poly(A)+ mRNA was taken from procyclic trypanosomes heated to 41°C for 1h; wild-type trypanosomes compared with parasites with *ZC3H11* RNAi. Other details as in (A).
