# Supplementary material for: Post-Transcriptional Regulation of the Trypanosome Heat Shock Response by a Zinc Finger Protein
Source: PLoS Pathog. 2013 Apr 4;9(4):e1003286. doi: 10.1371/journal.ppat.1003286 (PMC3616968; doi:10.1371/journal.ppat.1003286)
Supplement: Figure S4 — CAT reporter mRNAs. A. Constructs used to express CAT with various 3′-UTRs. All constructs are designed for integration in the beta tubulin locus. The CAT open reading frame (grey box) is preceded by the EP 5′ region and followed by a linker (in the control cell line) or the 3′-UTR or 3′-UTR fragment of interest (marked in light grey). This is followed by a truncated actin intergenic region still containing a possible poly(A) addition site (pA), the polypyrimidine tract (pY) and splice acceptor dinucleotide (AG). The expected reporter mRNA sizes (including the spliced leader sequence, without considering a poly(A) tail) are given below each construct. B. Northern blot with total RNA of CAT reporter cell lines and induction of ZC3H11 RNAi, hybridised with a CAT, ZC3H11 and HSP70 probes with 7SL used as a control. C. RNA from four different reporter cell lines (as indicated) was incubated with oligo d(T) and RNase H to remove poly(A) tails (Schwede et al, Nucleic Acids Res. 37, 5511–5528), before Northern blotting and hybridising with a CAT probe as in (B). The bands are of the expected sizes, D. AU-rich elements can effect reporter expression in a non-ZC3H11 dependent manner. Details as in Figure 4D. (PDF) [file ppat.1003286.s004.pdf]

A

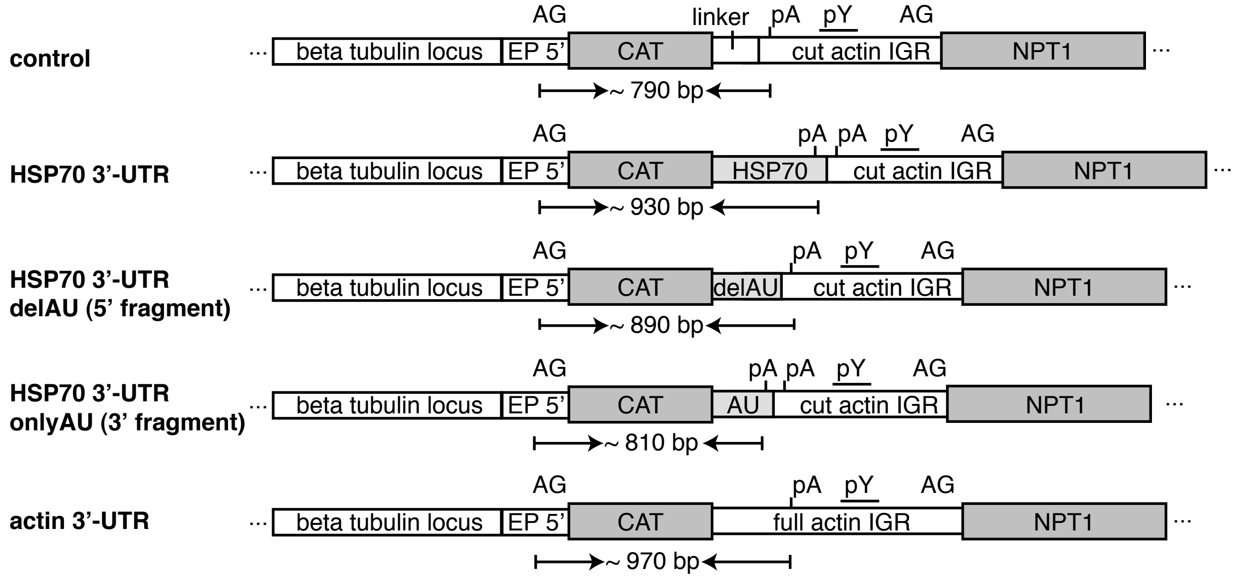

B

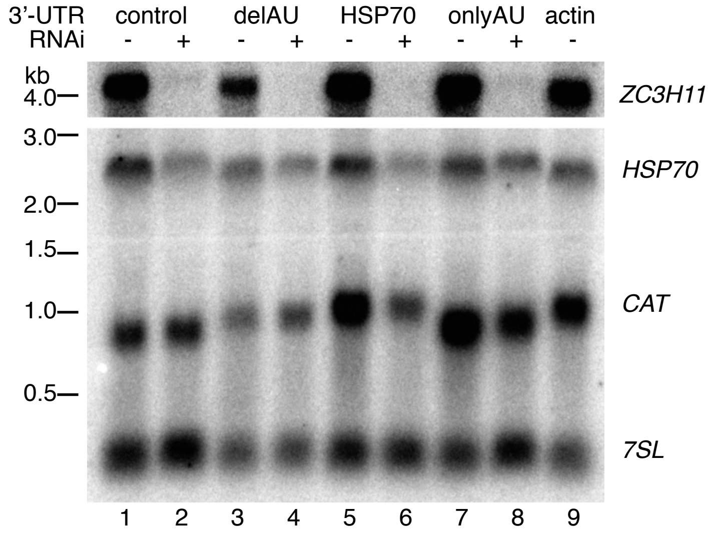

C

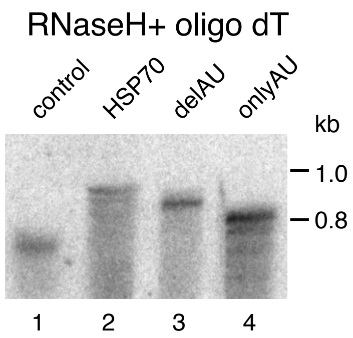

D

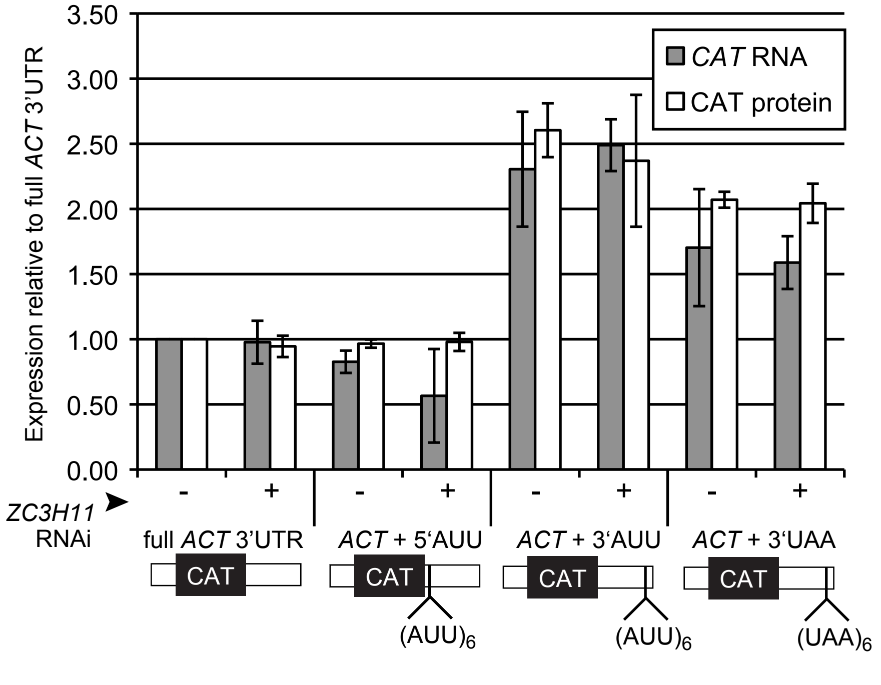

Supplementary Figure S4

CAT reporter mRNAs

A. Constructs used to express CAT with various 3'-UTRs.

All constructs are designed for integration in the beta tubulin locus. The *CAT* open reading frame (grey box) is preceded by the *EP* 5' region and followed by a linker (in the control cell line) or the 3'-UTR or 3'-UTR fragment of interest (marked in light grey). This is followed by a truncated actin intergenic region still containing a possible poly(A) addition site (pA), the polypyrimidine tract (pY) and splice acceptor dinucleotide (AG). The expected reporter mRNA sizes (including the spliced leader sequence, without considering a poly(A) tail) are given below each construct.

B. Northern blot with total RNA of CAT reporter cell lines and induction of *ZC3H11* RNAi, hybridised with a *CAT, ZC3H11* and *HSP70* probes with *7SL* used as a control.

C. RNA from four different reporter cell lines (as indicated) was incubated with oligo d(T) and RNase H to remove poly(A) tails (Schwede et al, Nucleic Acids Res. 37, 5511-5528) before Northern blotting and hybridising with a *CAT* probe as in (B). The bands are of the expected sizes.

D. AU-rich elements can affect reporter expression in a non-ZC3H11-dependent manner. Details are as in Figure 4D.
