# Supplementary material for: Post-Transcriptional Regulation of the Trypanosome Heat Shock Response by a Zinc Finger Protein
Source: PLoS Pathog. 2013 Apr 4;9(4):e1003286. doi: 10.1371/journal.ppat.1003286 (PMC3616968; doi:10.1371/journal.ppat.1003286)
Supplement: Figure S5 — Effects of heat shock (1 h, 41°C) on procyclic trypanosome mRNAs with or without ZC3H11 RNAi. Various blots from different experiments are shown, with quantitation relative to rRNA (marked with *) or the 7SL RNA. A. ZC3H11, HSP70 and HSP83 mRNA levels from the same experiment were quantified relative to the rRNA (methylene blue staining). B. HSP100 (Tb927.2.5980) and DNAJ2 (Tb927.2.5160) C. HSP110 (Tb927.10.12710) D. FKBP (Tb927.10.16100) E. Alpha tubulin, TUB F. Glycerol-3 phosphate dehydrogenase (GPDH, Tb927.8.3530). Due to a transfer problem at the 7SL signal, ribosomal RNA was used for normalization. (PDF) [file ppat.1003286.s005.pdf]

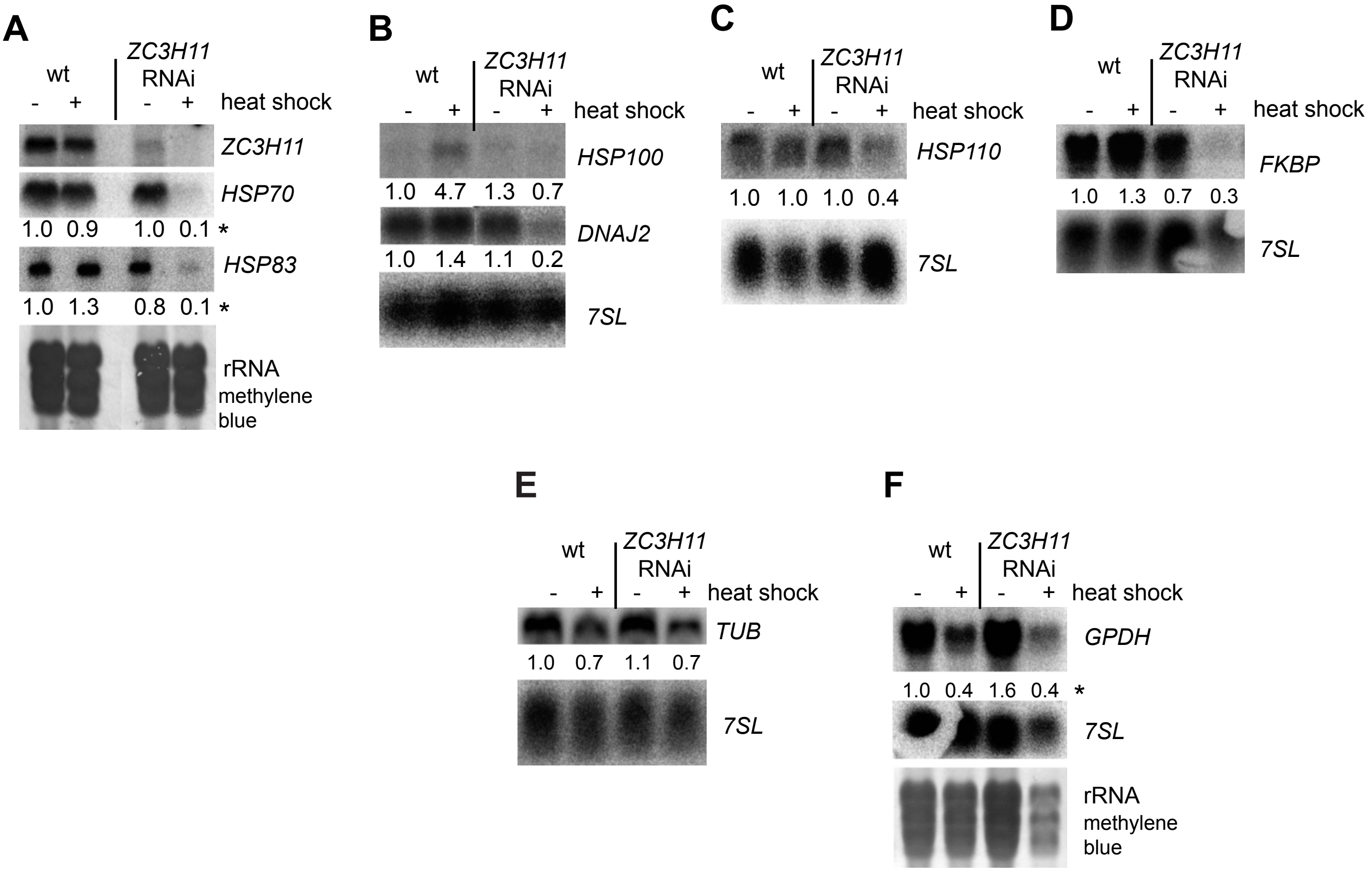

<u>Supplementary Figure S5</u>

Effects of heat shock (1h, 41°C) on procyclic trypanosome mRNAs with or without *ZC3H11* RNAi. Various blots from different experiments are shown, with quantitation relative to rRNA (marked with *) or the *7SL* RNA.

A. *ZC3H11, HSP70* and *HSP83* mRNA levels from the same experiment were quantified relative to the rRNA (methylene blue staining).

B. *HSP100* (Tb927.2.5980) and DNAJ2 (Tb927.2.5160)

C. *HSP110* (Tb927.10.12710)

D. FKBP (Tb927.10.16100)

E. Tubulin, *TUB*

F. Glycerol-3 phosphate dehydrogenase (*GPDH*, Tb927.8.3530). Due to a transfer problem at the 7SL signal, ribosomal RNA was used for normalization.
