# Supplementary material for: Post-Transcriptional Regulation of the Trypanosome Heat Shock Response by a Zinc Finger Protein
Source: PLoS Pathog. 2013 Apr 4;9(4):e1003286. doi: 10.1371/journal.ppat.1003286 (PMC3616968; doi:10.1371/journal.ppat.1003286)
Supplement: Table S2 — Plasmids and oligonucleotides used in this paper. (DOCX) [file ppat.1003286.s007.docx]

**Supplementary Table S2 Plasmids and oligonucleotides**

| **A. Plasmids used in this study** | |  |  |
| --- | --- | --- | --- |
| **Plasmid no.** | **description** | **Primers used for cloning** |  |
| ***Ectopic inducible expression from rDNA locus*** | |  |  |
| pHD1988 | stemloop RNAi targeting ZC3H11 | cz3149; cz3150 |  |
| pHD1950 | ZC3H11-TAP | cz3155; cz3456 |  |
| pHD2067 | ZC3H11-myc | cz3155; cz3456 |  |
| pHD2068 | N-terminal fragment of ZC3H11-myc (128aa up to first BamHI site) |  |  |
| pHD2299 | λN-ZC3H11-myc | cz4480; cz3456 |  |
| pHD2314 | λN-C-termZC3H11-myc (starts at I101) | cz4581; cz3456 |  |
| pHD2318 | λN-N-termZC3H11-myc (128aa, BamHI digestion of pHD2299) |  |  |
| pHD2219 | C70S mutant ZC3H11-myc (site directed mutagenesis) | cz4041; cz4042 |  |
| pHD1947 | in situ V5-tagged ZC3H11 | cz3164 to cz3167 |  |
| ***CAT reporter constructs constitutively expressed from the tubulin locus (Pol2)*** | |  |  |
| pHD2169 | CAT reporter, EP1 5'-UTR, 3' truncated actin IGR |  |  |
| pHD2239 | CAT reporter, HSP70 5'-UTR, 3' truncated actin IGR | cz3869; cz143 |  |
| pHD2240 | CAT reporter, EP1 5'-UTR, HSP70 3'-UTR | cz139; cz3868 |  |
| pHD2241 | CAT reporter, HSP70 5'-UTR, HSP70 3'-UTR | cz3869; cz143; cz139; cz3868 |  |
| pHD2272 | CAT reporter, EP1 5'-UTR, complete actin IGR (BamHI/SalI, from pHD1991) | | |
| pHD2270 | CAT reporter, EP1 5'-UTR, 5' part of HSP70 3'-UTR | cz139; cz4126 |  |
| pHD2271 | CAT reporter, EP1 5'-UTR, 3' part of HSP70 3'-UTR | cz4235; cz3868 |  |
| ***CAT reporter constructs constitutively expressed from the rDNA locus (Pol1)*** | |  |  |
| pHD2277 | CAT reporter for tethering, EP 5'-UTR and with 5x boxB before actin 3'-UTR | Mol Microbiol 83, 1048-63 |  |
| pHD1991 | CAT reporter for tethering, EP 5'-UTR and without boxB before actin 3'-UTR | Mol Microbiol 83, 1048-63 |  |
| ***ZC3H11 protein expression plasmids*** | |  |  |
| pHD2352 | TbZC3H11 104aa--N-terminal fragment cloned in pQEA38 | cz4595: cz4596 |  |
| pHD2353 | TbZC3H11 119aa--N-terminal fragment cloned in pQEA38 | cz4595; cz4595 |  |
| pHD2354 | TbZC3H11 136aa--N-terminal fragment cloned in pQEA38 | cz4595; cz4653 |  |
| pHD2355 | TbZC3H11 199aa--N-terminal fragment cloned in pQEA38 | cz4595; cz4652 |  |
| pHD2222 | *Tb*ZC3H11 full-length ORF cloned in pET-trx1b |  |  |
| pHD2388 | TbZC3H11 104aa--N-terminal C->S fragment cloned in pQEA38 | cz4595: cz4596 |  |
| pHD2389 | TbZC3H11 119aa--N-terminal C->S fragment cloned in pQEA38 | cz4595; cz4655 |  |

| **B. Oligonucleotides** | | | |
| --- | --- | --- | --- |
| **Oligo no.** | **Description** |  | |
| ***Oligonucleotides used for probes (Northern blot)*** | | **Sequence** | |
| cz2827 | HSP70 (Tb11.01.3110) probe FW | ggggatccATGACATACGAAGGCGCC | |
| cz3257 | HSP70 (Tb11.01.3110) probe RV | ACGACTCCGCAACCTCCT | |
| cz3135 | ZC3H11 probe FW | ACATCGTTCCAGAAACTGTGG | |
| cz3136 | ZC3H11 probe RV | TATCCGGACTCATTACCAACG | |
| cz2697 | CAT probe FW | TACACCGTTTTCCATGAGCA | |
| cz2698 | CAT probe RV | CCTGCCACTCATCGCAGTA | |
| cz4577 | HSP83 probe FW | TATTGTGAAGAAGGCCCTGG | |
| cz4578 | HSP83 probe RV | CTCTTTCATTGCCTTGCACA | |
| cz2581 | alpha tubulin probe FW | CCTTTGGCACAACGTCACCACGG | |
| cz2724 | alpha tubulin probe RV | TGACTCGCCGCAACCTCGAT | |
| cz4778 | HSP100 (Tb927.2.5980) probe FW | ACCTGAGCTCATCAATCGCT | |
| cz4779 | HSP100 (Tb927.2.5980) probe RV | CGTCAGTTTGTTCCCACCTT | |
| cz4776 | HSP110 (Tb927.10.12710) probe FW | GTGACGAACGAAGTCAGCAA | |
| cz4777 | HSP110 (Tb927.10.12710) probe RV | TGCATGAGCAACTTCCTTTG | |
| cz4788 | DnaJ2 (Tb927.2.5160) probe FW | TGTGGAGAAAAGGAGACGCT | |
| cz4789 | DnaJ2 (Tb927.2.5160) probe RV | ATCTTAACGCCACGACCATC | |
| cz4575 | PPIase FKBP (Tb927.10.16100) probe FW | GCCTCTCAACTATGCGGAAG | |
| cz4576 | PPIase FKBP (Tb927.10.16100) probe RV | AACAATGCCTTTGCGTTACC | |
| cz4632 | GPDH (Tb927.8.3530) probe FW | GAAGCGCGGTGCGGTG | |
| cz4633 | GPDH (Tb927.8.3530) probe FW | GTTGATGTCGGCCGCGG | |
| cz4490 | spliced leader oligo (anti sense) | CAATATAGTACAGAAACTGTTCTAATAATAGCGTTAGTT | |
|  | 7SL from pBS plasmid (T7/T3) |  | |
| ***Oligonucleotides used for cloning*** | | **Sequence** | **Rest-riction** |
| cz3149 | ZC3H11 stem loop fragment FW (pHD1988) | gagaagatctgcatgcATGAGCACTGCAACATCTGC | BglII; SphI |
| cz3150 | ZC3H11 stem loop fragment RV (pHD1988) | cggaattcgtcgacATCACACCTCTACGGTTGGC | EcoRI; SalI |
| cz3869 | HSP70 5'-UTR FW (pHD2239) | gacggatccGATAATGAGCGTTAGTGCT | BamHI |
| cz143 | HSP70 5'-UTR RV (pHD2239) | ctagaagcttCAAAGAGGCAGATATTCC | HindIII |
| cz139 | HSP70 3'-UTR FW (pHD2240, pHD2241 and pHD2270) | ctacggatccCAGGTGTATTTCGGACCGGTGTTGCAGT | BamHI |
| cz3868 | HSP70 3'-UTR RV (pHD2240, pHD2241 and pHD2271) | gagtcgacATAATGACGGTGGTGGGGAAC | SalI |
| cz4126 | 5' part of HSP70 3'-UTR RV (pHD2270) | gacgtcgacTAGTAGTAGCATCAATGTGTC | SalI |
| cz4235 | 3' part of HSP70 3'-UTR FW (pHD2271) | gacggatccGACACATTGATGCTACTAC | BamHI |
| cz3155 | ZC3H11 ORF FW (pHD1950, pHD2067) | taataagcttATGAGCACTGCAACATCTGC | HindIII |
| cz3456 | ZC3H11 ORF RV (pHD1950, pHD2067, pHD2299, pHD2314) | gacgttaacCAAGGAAAGAAACATATGCAGACC | HpaI |
| cz4480 | ZC3H11 ORF FW (pHD2299) | tatgggcccAGCACTGCAACATCTG | ApaI |
| cz4581 | ZC3H11 C-term. fragment FW (pHD2314) | tatgggcccATCATGGATGGACTCGTGACG | ApaI |
| cz4041 | ZC3H11 site directed mutagenesis FW (pHD2219) | ACAAAGCTGT**ct**AAAAACTTCGTG | |
| cz4042 | ZC3H11 site directed mutagenesis RV (pHD2219) | CTTGTAGCGCTCCGCCAAG | |
| cz3164 | ZC3H11 ORF fragment FW (pHD1947) | taatctcgagAGCACTGCAACATCTGCACC | XhoI |
| cz3165 | ZC3H11 ORF fragment RV (pHD1947) | atatgggcccGTTTCTGGAACGATGTAATCGC | ApaI |
| cz3166 | ZC3H11 5'-UTR fragment FW (pHD1947) | taatccgcggGTTTATAGAATAAAGCGGACTCG | SacII |
| cz3167 | ZC3H11 5'-UTR fragment RV (pHD1947) | atattctagaCACGTGAATAAACTCTCTGG | XbaI |
| cz4068 | ZC3H11 ORF FW | tggaattcATGAGCACTGCAAC | EcoRI |
| cz4069 | ZC3H11 ORF RV | atgtcgacCCAAGGAAAGAAAC | SalI |
| cz4074 | ZC3H11 ORF RV | cgctcgagTCACAAGGAAAGAAAC | XhoI |
| cz4524 | ZC3H11- N-term. ORF fragment RV | cgctcgagGGATCCTGACGAGGCC | XhoI |
| cz4525 | ZC3H11- C-term. ORF fragment FW | tggaattcATCATGGATGGACTCGTG | EcoRI |
| cz4595 | ZC3H11- N-term. ORF fragment FW (pHD2352, pHD2353, pHD2354, pHD2355) | attggtaccAGCACTGCAACATCTGCACC | KpnI |
| cz4596 | ZC3H11- N-term. ORF fragment RV (pHD2352) | gccaagcttATCCATCCATGATGTTCATCTC | HindIII |
| cz4652 | ZC3H11- N-term. ORF fragment RV (pHD2355) | gctaagcttAGGGATTATGTCGGACACAGC | HindIII |
| cz4653 | ZC3H11- N-term. ORF fragment RV (pHD2354) | gccaagcttAGGCAACATGAGATGAGTGATG | HindIII |
| cz4655 | ZC3H11- N-term. ORF fragment RV (pHD2353) | gccaagcttAATGCCACAGTTTCTGGAACG | HindIII |
